# Structure-oriented conversions of plastics to carbon nanomaterials

**DOI:** 10.1007/s44246-022-00016-2

**Published:** 2022-08-02

**Authors:** Shiying Ren, Xin Xu, Kunsheng Hu, Wenjie Tian, Xiaoguang Duan, Jiabao Yi, Shaobin Wang

**Affiliations:** 1 School of Chemical Engineering and Advanced Materials, The University of Adelaide, Adelaide, SA 5005 Australia; 2 Global Innovative Center for Advanced Nanomaterials, the University of Newcastle, Callaghan, NSW 2308 Australia

**Keywords:** Plastics, Carbon nanomaterials, Pyrolysis, Carbon recycle, Transformation

## Abstract

The accumulation of waste plastics has caused serious environmental issues due to their unbiodegradable nature and hazardous additives. Converting waste plastics to different carbon nanomaterials (CNMs) is a promising approach to minimize plastic pollution and realize advanced manufacturing of CNMs. The reported plastic-derived carbons include carbon filaments (i.e. carbon nanotubes and carbon nanofibers), graphene, carbon nanosheets, carbon sphere, and porous carbon. In this review, we present the influences of different intrinsic structures of plastics on the pyrolysis intermediates. We also reveal that non-charring plastics are prone to being pyrolyzed into light hydrocarbons while charring plastics are prone to being pyrolyzed into aromatics. Subsequently, light hydrocarbons favor to form graphite while aromatics are inclined to form amorphous carbon during the carbon formation process. In addition, the conversion tendency of different plastics into various morphologies of carbon is concluded. We also discuss other impact factors during the transformation process, including catalysts, temperature, processing duration and templates, and reveal how to obtain different morphological CNMs from plastics. Finally, current technology limitations and perspectives are presented to provide future research directions in effective plastic conversion and advanced CNM synthesis.

## Highlights

The impact factors in transforming plastics into carbon nanomaterials are reviewed.

The carbon morphology tendency from different plastics is revealed.

Directions for future research on plastic carbonization are presented.

## Introduction

Since the first synthesis of Bakelite in 1907 (Thompson et al. 2009), plastics have attracted extensive interest due to their outstanding properties such as lightweight, stability, flexibility, and low production costs. Various types of plastics have been explored for different applications. Moreover, as a result of the rapid increase in population and wide usage in different fields, global plastics production has soared to 367 million tons in 2020 from 1.5 million tons in 1950 (PlasticsEurope 2021). Also, the outbreak of COVID-19 called for a vast production of plastic personal protective equipment (including masks, gloves, and protective clothing). The increase of packaged take-out meals and home-delivery groceries caused by lockdown also resulted in a massive accumulation of disposable plastics (Adyel 2020). Due to the resistance to biodegradation and soaring production of plastics, the proliferation of waste plastics is becoming one of the most serious environmental problems, known as “white pollution”.

Plastic recycling is a sustainable approach to reduce pollution, but the requirement for plastic separation and purification increases its operating costs. Tertiary treatment can recover energy from plastic incineration. However, the massive emission of CO_2_ will worsen the global warming problem. In addition, the majority of plastics still end up in landfills (e.g., 23.4% of collected plastics in Europe were landfilled in 2020). Recently, a polyethylene terephthalate (PET) hydrolase designed by machine learning algorithm showed a superior behavior that can degrade PET bottles in two weeks. The obtained terephthalic acid has high purity and can be repolymerized to PET, realizing a close loop of plastic recycling (Lu et al. 2022). However, biodegradation of other plastics still suffers suffering from low efficiencies. Advanced oxidation processes have high efficiencies in degrading various plastics (Hu et al. 2022; Kang et al. 2019), but the reutilization of carbon elements in intermediates needs further improvements. Therefore, it is more favorable to decompose plastics to alleviate plastic pollution and fully reutilize the carbon sources to develop other value-added products.

Carbon nanomaterials (CNMs) have been applied as catalysts and adsorbents in environmental and energy sectors due to their physical properties, such as high mechanical strength, and good electrical and thermal conductivity (Azara et al. 2022). Traditionally, CNMs are produced from hydrocarbon gases (e.g., methane, ethylene, and propylene). However, the commercialization of CNMs is limited because these feedstocks are expensive and produced from non-renewable fossil fuels. As plastics are low-cost and carbon-rich products, they are ideal precursors for synthesizing functional CNMs, such as carbon nanotubes (CNTs), graphene, and nanocomposites (Zhuo and Levendis 2014). Therefore, using plastics to synthesize CNMs will decrease the manufacturing cost and accelerate the commercialization of CNMs in diverse applications. Other advantages of using plastics as CNM precursors include their processability and controllable weight. Plastics can be pre-formed according to different requirements, and the amount of carbon input can be accurately adjusted by controlling the precursor weight (Gong et al. 2019). Thus, converting plastics to high-value-added carbon is a promising way to transform waste into wealth.

Recently, some reviews on plastics transformation to CNMs have been reported, but they did not encompass all types of carbon products. For examples, Zhang et al. (Zhang et al. 2021b) and Williams et al. (Williams et al. 2021) discussed hydrogen and CNTs production from plastics and Azara et al. (Azara et al. 2022) mainly focused on carbon filaments, while Vieira et al. (Vieira et al. 2022) only reviewed graphene-based materials. Although Gong et al. (Gong et al. 2019) have summarized the carbonization of general polymers, the impact factors including plastic types were not comprehensively reviewed. Thus, to provide inspiration for future research to obtain desired carbon products, a comprehensive review of the influence of plastic types on morphology control of carbon products is required. In addition, the impact factors on the morphological control of CNMs from plastics also need to be comprehensively clarified. For example, two steps are generally involved in the CNM synthesis via carbonization: pyrolysis and catalysis (carbon formation); however, the impacts of pyrolysis on the second step are unclear. The preferred carbonization conditions to obtain specific morphologies are still vague and have not been reported.

In this review, features of different types of plastics will be firstly introduced. Then the focus will be on the formation of pyrolysis intermediates with different types of plastics feedstocks and the influences of intermediates on the formation of different carbon morphologies, including CNTs, graphene, carbon nanosheets (CNS), carbon sphere (CS), and porous carbon. Later, we will summarize other critical factors of plastic carbonization, such as catalysts, reactors, temperature, and duration, and further discuss their impacts on the qualities and yields of CNMs. On this basis, effective approaches to accurately controlling the shape and structure of CNMs will be summarized, which can provide guidance for plastic carbonization in future research. At last, perspectives and directions of plastic carbonization for future research will be pictured.

## Impacts of plastic types on the plastics carbonization

**Fig. 1:**
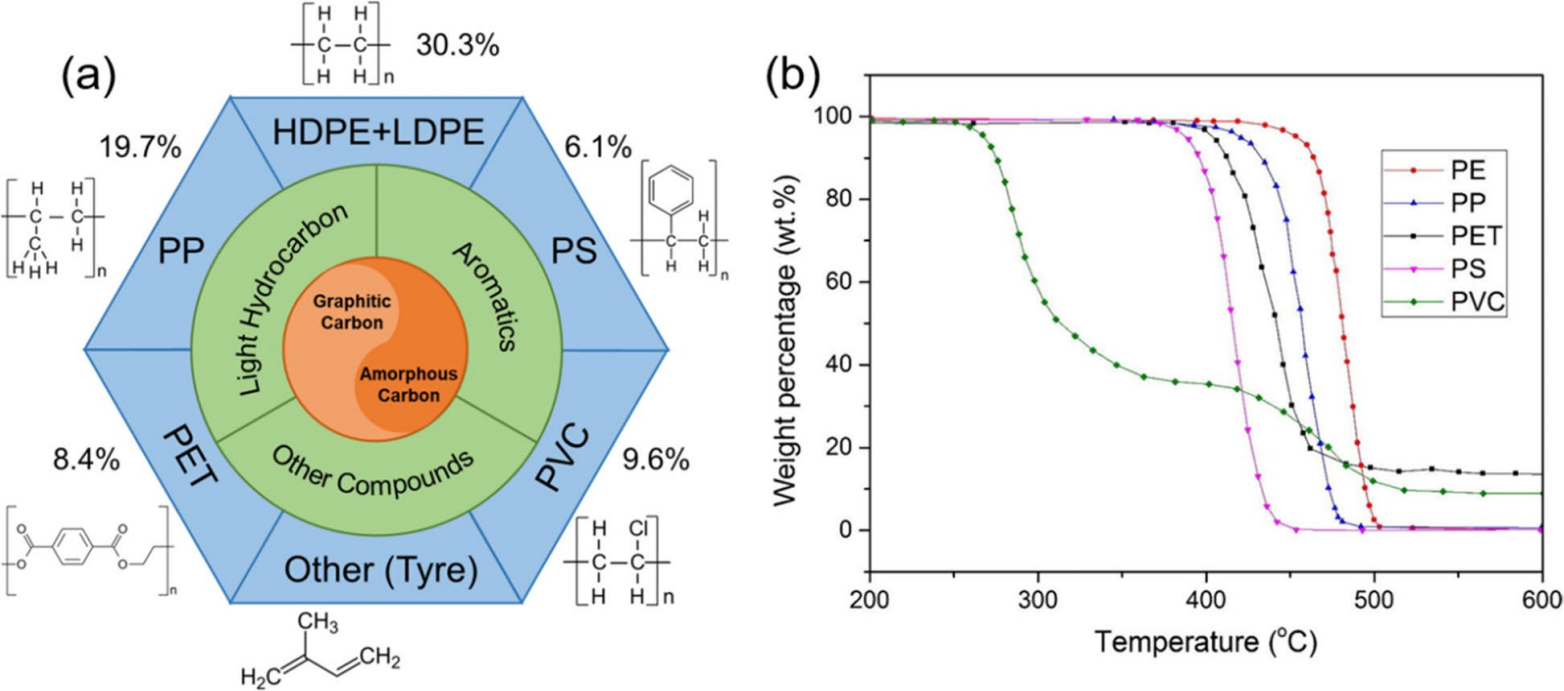
**a** The relationship between plastics and carbon category. The percentage represents the demand of each kind of plastics in Europe in 2020, reproduced with permission from PlasticsEurope, ( 2021); **b** Thermogravimetric analysis (TGA) of different plastics, conditions: PE, PP, PS, and PVC were pyrolyzed under nitrogen, PET was pyrolyzed under helium, and the heating rate is 10 °C min^− 1^, reproduced with permission from Yu et al., (Yu et al. 2016), copyright 2016 Elsevier

Plastics are ubiquitous in the environment. They play a vital role in many industries, such as packaging, construction, and textiles, etc. The commonly used plastics include polyethylene (PE), polypropylene (PP), polystyrene (PS), PET, polyvinyl chloride (PVC), and more. The molecular structure of these plastics and their demand in Europe in 2020 are illustrated in Fig. 1a.

Plastics are considered carbon-rich sources, but different plastics possess distinct structures, leading to different pyrolysis temperatures for transformation. As shown in Fig. 1b, there are two stages in PVC pyrolysis. The first stage occurs at 250–350 °C with about 65% weight loss, and the main reaction in this stage is dehydrochlorination. During the second stage at 350–525 °C, cracking and decomposition of de-HCl PVC occur. However, the pyrolysis of other plastics only has one stage and their pyrolysis temperatures are distinct, which might be due to their different chain compositions and structural stability. The stability sequence of different plastics can be concluded as PE > PP > PET > PS > PVC. In addition, structural differences affect pyrolysis products, which will impact the subsequent carbon formation. In this session, the effects of plastic compositions on the carbonization process will be discussed. The contents of different major plastics are summarized in Table 1.

**Table 1:** Compositions of different plastics (Panahi et al. 2019)

Plastics	Molecular Formula	Carbon (wt%)	Hydrogen (wt%)	Oxygen (wt%)	Chlorine (wt%)
PE	(C_2_H_4_)_n_	85.7	14.4	–	–
PP	(C_3_H_6_)_n_	85.7	14.3	–	–
PS	(C_8_H_8_)_n_	92.3	7.7	–	–
PET	(C_10_H_8_O_4_)_n_	62.5	4.2	33.3	–
PVC	(C_2_H_3_Cl)_n_	38.4	4.8	–	56.8

### PE

PE is a polymer of ethylene with a long chain structure. There are mainly three types of PE, which are high-density polyethylene (HDPE), low-density polyethylene (LDPE), and linear low-density polyethylene (LLDPE). HDPE possesses higher strength and hardness than LDPE and it is usually applied in rigid packaging (e.g., milk containers, detergent bottles, and bottle crates), while LDPE is widely used in bags, bottles, wrappers, and housewares. During the pyrolysis process, C-C bonds located in side chains are vulnerable, thus, thermal decomposition of LDPE is easier than HDPE and LLDPE due to a higher content of branches in LDPE. Compared to other plastics, more light hydrocarbons can be obtained from PE during pyrolysis, which facilitates the formation of CNTs because light hydrocarbons are the primary feedstock for CNTs synthesis (Cai et al. 2021). Therefore, PE is an excellent solid feedstock for CNTs production. In addition, light hydrocarbons are suitable precursors to form other carbon products. Algozeeb et al. (Algozeeb et al. 2020) transformed PE into flash graphene by a flash Joule heating method. Pol (Pol 2010) produced carbon spheres from PE under high-temperature and high-pressure conditions. Recently, Algozeeb et al. (Algozeeb et al. 2020) successfully produced porous carbons from the mixture of HDPE and potassium acetate (KOAc).

### PP

PP has similar properties to PE but with greater hardness and heat resistance. PP is the second-most produced plastic and is widely used in furniture, clothing, masks, and plastic molding, etc. During pyrolysis, PP will be cracked into oligomers via random scission of C-C bonds, then further decomposed into small molecules such as hydrogen, methane, and propylene through beta scission (Vollmer et al. 2020). Similar to PE, PP is also an excellent feedstock to produce CNTs, graphene, and carbon spheres. Jia et al. (Jia et al. 2022) fabricated multi-wall carbon nanotubes from PP over A-site-deficient perovskite. Sawant et al. (Sawant et al. 2013) successfully synthesized carbon spheres from PP under high-temperature and high-pressure conditions. Gong et al. (Gong et al. 2014b) converted PP into graphene flakes using organically modified montmorillonite (OMMT) as a catalyst and template.

### PS

PS is an aromatic hydrocarbon polymer produced from monomer styrene and is widely applied in protective packaging, foams, and disposable cutlery, etc. Due to the phenyl group in structure, PS is prone to being pyrolyzed to aromatic hydrocarbons and oil compounds. Aromatics are mainly responsible for the formation of amorphous carbons (Cai et al. 2021). Thus, PS is not favorable for fabricating high-quality CNTs, but it can be converted to other carbon products. Cui et al. (Cui et al. 2017) obtained high-quality graphene foil from PS by the chemical vapor deposition (CVD) method. When templates are introduced during the carbonization process, aromatics tend to form carbon nanosheets on the templates, giving PS higher yields of carbon nanosheets than other plastics (Hong et al. 2012).

### PET

The structure of PET has repeating units of ethylene terephthalate. It is widely used in textiles, water bottles, cosmetic jars, and plastic tapes, etc. The oxygen content in PET is relatively high (around 33.3 wt%), so CO_2_ is the main gas product during pyrolysis. Only limited carbons in PET could be transformed into solid carbonaceous materials (Yao et al. 2022). Similar to PS, PET also contains benzene rings in its structure, resulting in a higher aromatic content after pyrolysis (Zhuo et al. 2012). However, PET can be transformed into porous carbon under activation. Zhang et al. (Zhang et al. 2021a) synthesized hierarchical porous carbon from PET with the activation of ZnCl_2_/KOH. Wen et al. (Wen et al. 2020) converted PET into porous carbon nanosheets using OMMT as a catalyst and template while KOH was used as the activator.

### PVC

PVC has a linear structure with chlorines on alternating carbon centers. It is often used in drainage pipes, medical devices, and resilient flooring. Compared to other plastics, PVC has the lowest carbon content and generates HCl during pyrolysis, so it is not an ideal feedstock for carbonization to synthesize CNMs other than porous carbon. Cheng et al. (Cheng et al. 2015) transformed PVC into nanoporous carbon using Mg(OH)_2_ as a hard template. In addition, adding a small amount of PVC to LLDPE can promote the formation of CNTs because the chlorine radicals decomposed from PVC will accelerate the dehydrogenation and aromatization of LLDPE. However, excess chlorine radicals will poison catalysts and hamper the formation of CNTs (Gong et al. 2013d).

### Other plastics

Apart from the above plastics, tyres are also used to fabricate carbonaceous materials. Tyres are polymer hybrids and can be pyrolyzed into a wide range of aliphatic and aromatic compounds. Zhang et al. (Zhang et al. 2022) found that, rather than the aliphatic compounds (hexadecane and decane), aromatic compounds (styrene, naphthalene, and phenanthrene) pyrolyzed from tyres were responsible for the formation of solid carbon nanomaterials. Apart from virgin plastics, mixed waste plastics can also be utilized to produce carbonaceous materials, but they always contain contaminants such as S, N, P, and Cl, which might promote the cracking of C-C bonds or the aromatization of aliphatic compounds. Consequently, waste plastics can obtain higher gas and pyrolysis oil yields than virgin plastics after pyrolysis (Borsodi et al. 2016).

In general, the category of plastics has a great influence on carbon formation. The distinct structures make plastics tend to generate different intermediates during the pyrolysis process. According to the pyrolysis reactions at the polymer backbone, the above-mentioned plastics can be divided into two categories, non-charring and charring plastics (Gong et al. 2019). During the pyrolysis process, the backbones of non-charring plastics (e.g., PE and PP) prefer to be decomposed into light hydrocarbons (e.g., ethylene, propylene, and single ring benzene), which are favorable feedstocks of carbon filaments, graphene, and carbon spheres. On the other hand, the backbone of charring plastics (including PET, PVC, and PS) experiences cyclization, aromatization, and crosslinking instead of being decomposed into small molecular gases, and then carbon material frames will be constructed. This is beneficial for the production of carbon nanosheets and porous carbon but is detrimental to the formation of smooth carbon spheres (Algozeeb et al. 2022; Sawant et al. 2013). Most waste plastics are mixed plastics and consist of additives and contaminants, so the influences of the three factors should be critical for the carbonization and reutilization of plastics, and deserve more attention in future research.

## Morphology control of carbonaceous materials

Many investigations have focused on the morphology control of plastic-derived carbonaceous materials (Azara et al. 2022; Bazargan and McKay 2012; Choi et al. 2022; Harussani et al. 2022; Williams 2021; Zhang et al. 2021b). CNTs, carbon nanofibers (CNFs), graphene, CS, CNS, and porous carbon have been successfully synthesized from various plastics (Deng et al. 2016; Gong et al. 2019; Vieira et al. 2022). Generally, the conversion of plastics to CNMs experiences two steps: pyrolysis and catalysis (carbon formation). During the pyrolysis process, plastics will be broken down into hydrogen, light hydrocarbons, aromatics, and liquid oils (Gong et al. 2012b; Xu et al. 2021a). Subsequently, these small molecules will act as the precursors for CNMs formation with the presence of catalysts (Gong et al. 2013a; Gong et al. 2014c; Wei et al. 2011; Zhou et al. 2022a). In this section, detailed information on the formation of different morphological carbon products will be summarized.

### Carbon filaments

Carbon filaments can be divided into CNTs and CNFs. Compared with CNFs, CNTs possess a hollow structure and thus a higher surface-to-volume area. As high-value carbon products, carbon filaments have remarkable mechanical, electrical, and thermal properties, so plenty of efforts have been made to control the morphologies and promote their performance (Azara et al. 2022). The morphology of carbon filaments is closely related to the nature and the particle size of metal catalysts (Jiang et al. 2007; Moisala et al. 2003). Hence, preparing an effective catalyst with a suitable size and crystalline structure is crucial for the growth of on-demand carbon filaments.

**Fig. 2:**
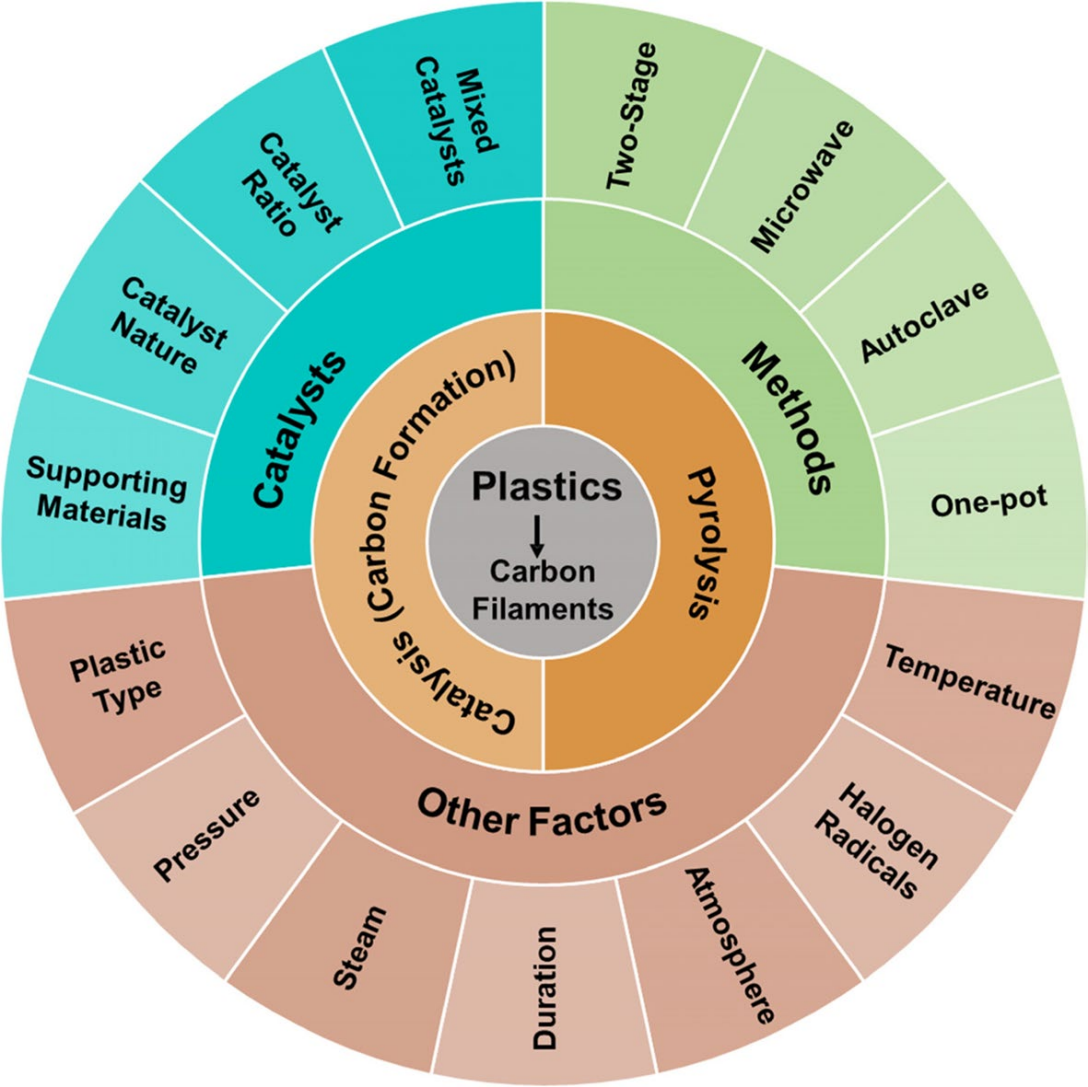
The impact factors related to the conversion of plastics to carbon filaments

Factors that impact the yields, morphology, and quality of carbon filaments from plastics have been summarized in Fig. 2. During the pyrolysis process, catalysts (ratio and type), plastic types, temperature, and halogen elements all impact the compositions of decomposed products such as light hydrocarbons and aromatics, which are the major feedstock for developing carbon filaments (Gong et al. 2013a; Gong et al. 2014c; Yang et al. 2016; Yang et al. 2020). During the formation of carbon filaments, catalysts, temperature, and carbonization methods are the three decisive factors. Other factors such as steam, pressure, atmosphere, and duration also influence the quality and yields of carbon filaments. Details about the impact factors related to carbon filament synthesis are summarized in Table 2.

**Table 2:** The impact factors related to the conversion of plastics to carbon filaments

Impact factor			Main point	Reference
Pyrolysis	Catalysts	Types	Zeolites (ZSM-5, MCM-41, SBA-15, etc.) are effective catalysts for the pyrolysis of different plastics to obtain more light hydrocarbons and aromatics. The ratio of Si/Al in zeolites is a vital factor influencing gas product composition. Additionally, catalysts such as activated carbon, carbon black, and biomass can promote gaseous yields for filament formation.	Gong et al. 2013a, Gong et al. 2012b; Liu et al. 2011; Thivasasith et al. 2022; Wen et al. 2014; Xu et al. 2021a, Xu et al. 2021b, Xu et al. 2020; Yang et al. 2020, Yang et al. 2016; Yao et al. 2021b, Yao et al. 2018a
		Ratio	The content of light hydrocarbons and aromatics will increase along with the higher ratio of catalysts to plastics.	Acomb et al. 2015; Elbaba and Williams 2012; Gong et al. 2013a
	Temperature		Increasing temperature can promote plastics pyrolysis and the generation of gaseous products, then enhancing carbon yields. The commonly used temperature range is between 500 ~ 600 °C.	Elbaba and Williams 2012; Liu et al. 2011; Wulan et al. 2018; Yao et al. 2021a
	Halogen radicals		A small amount of halogen radicals can facilitate the dehydrogenation and aromatization of certain plastics during the pyrolysis process, which is beneficial to the formation of CNTs. However, excessive halogen radicals will lead to low quantity and purity of CNTs.	Gong et al. 2014c, Gong et al. 2013d, Gong et al. 2012a; Wu et al. 2014; Yu et al. 2009
	Plastic types		During decomposition, plastic structures determine the compositions of gaseous intermediates. Compared with HDPE, PP and LDPE have more branches on the backbone, which facilitates the formation of light hydrocarbons and then the synthesis of CNTs. The pyrolysis gas yields from different plastics follow the sequence of LDPE > PP > HDPE > PET > PS, which is consistent with their CNTs yields. The high oxygen content in PET results in higher proportions of CO_2_ and CO instead of gaseous hydrocarbons. For PS, the existence of benzene rings leads to a higher portion of liquid oils.	Aboul-Enein et al. 2018; Acomb et al. 2014; Borsodi et al. 2016; Cai et al. 2021; Gong et al. 2014c; Panahi et al. 2019; Yao et al. 2022; Zhuo et al. 2012
Catalysis (carbon formation)	Catalysts	Types	Transition metal compounds such as metal oxides (NiO, Ni_2_O_3_, Fe_2_O_3_, Fe_3_O_4_, Co_3_O_4_, etc.) and ferrocene are frequently used to synthesize CNTs. In addition, biochar, nanoporous anodic alumina membrane, and red mud have been applied to promote the synthesis of CNTs.	Acomb et al. 2016; Altalhi et al. 2013; Gong et al. 2014c, Gong et al. 2013d; Huang et al. 2021; Liu et al. 2011; Shah et al. 2022; Wang et al. 2020; White et al., 2021; Xu et al. 2021c; Zhang et al. 2022
		Ratio of catalyst to plastic	A rational ratio of catalysts to plastics is required to optimize the formation of light hydrocarbons. A too high or too low ratio will cause fewer CNT yields or excess unreacted plastics, respectively.	Acomb et al. 2015; Elbaba and Williams 2012; Xu et al. 2021a, Xu et al. 2021b, Xu et al. 2020
		Mixed catalysts	Various kinds of metallic compounds can be mixed to accelerate the formation of CNTs, such as Ni, Co, Fe, Al, Mg, Mn, Mo, and stainless steel.	Aboul-Enein and Awadallah 2018; Haggar et al. 2022; Jia et al. 2022; Panahi et al. 2019; Ribeiro et al. 2022; Veksha et al. 2022; Wu et al. 2014; Yang et al. 2015; Yao et al. 2022, Yao et al. 2021a
		Supporting materials	Supporting materials are commonly combined with transition metals to alleviate the coalescence and sintering of catalysts and then facilitate the formation of CNTs. Many compounds have been used as supporting materials, including CaO, SiO_2_, zeolite, CuBr, Al_2_O_3_, MgO, TiO_2_, cordierite, montmorillonite, citric acid, activated carbon, and char.	Gong et al. 2015b, Gong et al. 2012b; Modekwe et al. 2021; Ribeiro et al. 2022; Song et al. 2007; Thivasasith et al. 2022; Veksha et al. 2022; Wang et al. 2020; Wu and Williams 2009; Xu et al. 2021a; Yang et al. 2020, Yang et al. 2015; Yao et al. 2021b, Yao et al. 2018a, Yao et al. 2018b, Yao et al. 2017; Zhang et al. 2022
	Synthesis methods	Two-stage	Two-stage (pyrolysis and catalysis) method consists of two independent reactors which can be separately heated and controlled. It is the most frequently used approach to fabricate high-quality CNTs from plastics, and is suitable for continuous fabrication, but the carbon yield is usually below 20%.	Gangoli et al. 2022; Jia et al. 2022; Liu et al. 2011; Tripathi et al. 2017; Xu et al. 2021b; Yang et al. 2018; Yao et al. 2018b, Yao et al. 2017; Zhuo et al. 2010
		Microwave	Microwave is a rapid heating method and can effectively transform different plastics into hydrogen and CNTs in 30–90 seconds. The yields of carbon and hydrogen obtained from the microwave method can achieve 80% and 97%, respectively.	Jie et al. 2020; Sridhar and Park 2020
		Autoclave	This method has high requirements for device as it undergoes at high temperature and pressure. The highest yield of carbon synthesized by an autoclave is up to 80%.	Kong and Zhang 2007; Pol and Thackeray 2011
		One-pot	The one-pot method is facile to produce CNTs but unsuitable for scale-up production.	Gong et al. 2013d, Gong et al. 2013a, Gong et al. 2012a; Ha et al. 2022
	Atmosphere		The carbonization is usually conducted at an inert atmosphere (nitrogen or argon). Hydrogen atmosphere was applied to reduce catalyst precursors to metal catalysts, which will facilitate the formation of CNTs.	Li et al. 2019
	Pressure		The appropriate pressure (~ 1 MPa) added into the reactor facilitates to attain high-quality CNTs with high yields, but if the pressure is too high, the growth of CNTs will be inhibited.	Wang et al. 2020
	Temperature		The solubility of carbon in metallic catalysts will increase along with the temperature, which is conducive to the formation of carbon filaments. The regular temperature range for the formation of CNTs is between 700 ~ 900 °C, but the optimum temperature varies on the specific condition in experiments.	Acomb et al. 2015; Jia et al. 2022; Liu et al. 2011; Shah et al. 2022; Tripathi et al. 2017; Xu et al. 2021c; Yang et al. 2018; Zhang et al. 2017; Zhuo et al. 2010
	Time		The length of CNT arrays is dependent on reaction time. Longer CNTs can be obtained with increased retention time.	Yang et al. 2010
	Steam		Steam is often introduced to facilitate hydrogen and syngas yields, but the optimum injection amount varies between different investigations. In some tests, a low-level injection of steam can improve the quality and purity of CNTs.	Acomb et al. 2014; Elbaba and Williams 2012; Wu et al. 2014; Yao et al. 2018a, Yao et al. 2018b

#### Carbon nanotubes

Since the first report on CNTs in 1991 (Iijima 1991), CNTs have drawn widespread interests due to their outstanding physical, chemical, and mechanical properties. Carbon-containing gases (e.g., CH_4_, C_2_H_2_, CO, etc.) are conventional feedstocks for CNTs fabrication. However, these fossil fuel-derived precursors are expensive, which impedes the commercial applications of CNTs. The first report on conversion of solid polymers to CNTs was published in 1996 (Cho et al. 1996). Since then, numerous efforts have been devoted to transforming low-cost waste plastics into CNTs (Bazargan and McKay 2012). Until now, besides regular CNTs, different morphologies of CNTs, such as cup-stack, bamboo-like, and ultra-straight CNTs, have been successfully synthesized from plastics.

##### Regular CNTs

**Fig. 3:**
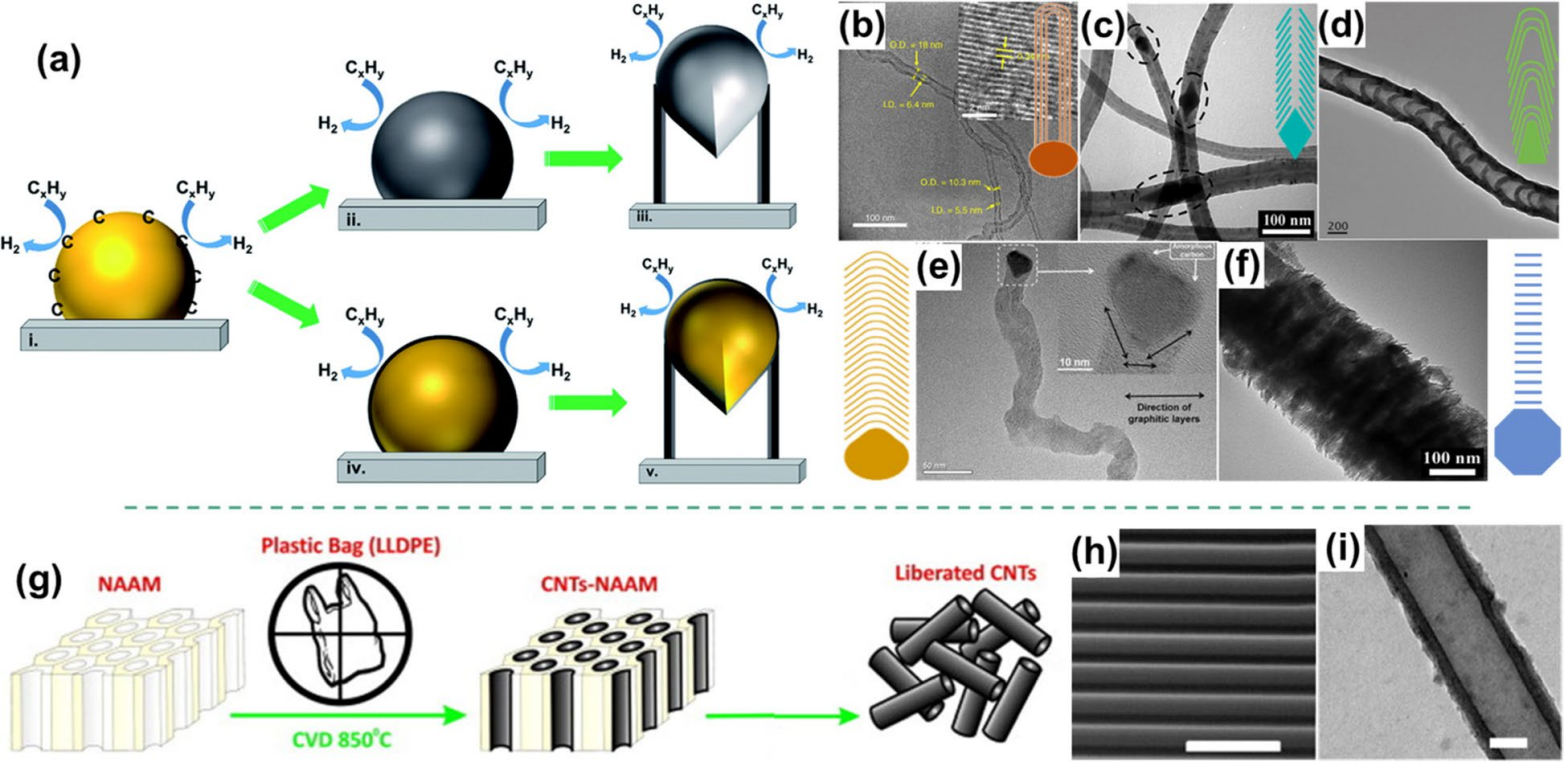
**a** The VLS and VSS mechanisms for the growth of carbon nanotubes, reproduced with permission from Tessonnier et al. (Tessonnier and Su 2011), copyright 2011 John Wiley and Sons; **b** A TEM image of multiwall CNTs synthesized by microwave with FeAlO_x_ catalysts, reproduced with permission from Jie et al. (Jie et al. 2020), copyright 2020 Springer Nature; **c** A TEM image of cup-stack CNT produced from PP by the catalysis of Ni with lattice oxygen; **d** A TEM image of bamboo-like CNT prepared from PP using nanosized biochar, reproduced with permission from Shah et al. (Shah et al. 2022), copyright 2021 Elsevier; **e** A TEM image of carbon nanofiber fabricated from PP by the catalysis of Ni/OMMT, reproduced with permission from Gong et al. (Gong et al. 2013b), copyright 2013 Elsevier; **f** A TEM image of PL-CNF produced from PP by the catalysis of Ni, reproduced with permission from Gong et al. (Gong et al. 2015b), copyright 2015 The Royal Society of Chemistry and the Centre National de la Recherche Scientifique; **g** The schematic diagram for the synthesis of well-organized CNTs by NAAM templates; **h** and **i** are the SEM (scale bar =250 nm) and TEM (scale bar =10 nm) images of CNTs synthesized by NAAM templates, reproduced with permission from Altalhi et al. (Altalhi et al. 2013), copyright 2013 Elsevier

Regular CNTs have a hollow structure, and the graphitic layers parallel their growth axis (Fig. 3b). Currently, the two-stage method with transition metal catalysts is believed to be the most popular way to produce CNTs from plastics. Microwave carbonization is a new efficient approach to transforming plastics into CNTs and hydrogen (Jie et al. 2020).

Previously, vapor-liquid-solid (VLS) growth mechanism of CNTs proposed by Baker et al. (Baker et al. 1972) was well accepted by researchers. Figure 3a shows the three stages in the VLS mechanism. At stage (I), the gaseous carbon precursors adsorb and dissociate into elementary carbon atoms on the surface of catalysts. At stage (II), carbon atoms are dissolved and diffused in the whole catalyst particle and generate liquid metastable carbides. At stage (III), carbon atoms accumulate at the catalyst fringe and form graphene layers, which will grow up into CNTs. However, some controversies concerning the diffusion step were raised because the driving force of carbon diffusion in the catalyst particle was still not clear. The VLS mechanism could explain the cases that catalyst particles are melted out in the diffusion step, but it is not valid when the particles remain solid. Therefore, Tessonnier et al. (Tessonnier and Su 2011) proposed a vapor-solid-solid (VSS) growth mechanism (Fig. 3a, IV and V). In the VSS mechanism, stages (II) and (III) are substituted by (IV) and (V), respectively. The carbon atoms only dissolve and diffuse on the surface of catalyst particles. Based on these mechanisms, numerous studies have been carried out to optimize the production of CNTs. All the factors that may impact the yield and quality of plastic-derived CNTs are summarized in Table 2.

##### Cup-stack CNTs

Unlike regular CNTs, the graphitic layers in cup-stack CNTs have a 17-25^o^ angle to their growth axis. This is mainly due to the rhombic shape of Ni catalysts (Gong et al. 2013b; Gong et al. 2015b). At the initial step, the graphene layers formed on the crystal facet are parallel to the crystal plane. There is a certain angle between the two facets of the rhombic-shaped catalyst, hence the graphene layers grow with the same angle and result in the formation of cup-stack CNTs (Fig. 3c). Therefore, controlling the shape of catalysts is the key to obtaining cup-stack CNTs. Gong et al. (Gong et al. 2015b; Gong et al. 2013b) found that combining OMMT or increasing lattice oxygen in a nickel catalyst could coalesce and reconstruct NiO nanoparticles into the rhombic shape. Subsequently, NiO nanoparticles are reduced into rhombic-shaped metallic Ni, which will catalyze the transformation of hydrocarbons and aromatics to cup-stack CNTs.

##### Bamboo-like CNTs

Bamboo-like CNTs consist of periodically separated segments of graphitic layers along the growth axis. Compared to other kinds of CNTs, they have a high defect density and specific surface areas because of their unique morphology. Shah et al. (Shah et al. 2022) adopted biochar as the catalyst and synthesized the bamboo-like CNTs with a uniform diameter. They found that the morphology of CNTs was mainly affected by the temperature and particle size of biochar. Large-size biochar favors to form amorphous carbon, and the nanosized biochar tends to produce high-quality bamboo-like CNTs. Meanwhile, raising the temperature will facilitate hydrocarbons decomposition and accelerate the diffusion rate of carbons. It is are beneficial for obtaining CNTs with a high quality and graphitization degree. In addition, catalysts with triangle or droplet shapes are prone to generating bamboo-like CNTs (Fig. 3d).

##### Ultra-straight CNTs

Altalhi et al. (Altalhi et al. 2013) have successfully transformed plastic bags into ultra-straight CNTs with smooth surfaces and open ends. As shown in Fig. 3g-i, nanoporous anodic alumina membranes (NAAMs) act as templates to control the length and outside diameter of CNTs, while the walls and inner diameter can be controlled by adjusting deposition time. Adding templates during carbonization is a facile way to control the morphology of CNTs, but relevant research is limited and further study is recommended.

#### Carbon nanofibers

Compared to CNTs, carbon nanofibers (CNFs) have a solid core. CNFs can be manufactured on a large scale and are cost-effective products, so they are excellent alternatives to CNTs (Azara et al. 2022). CNFs also have some different morphologies, such as regular CNFs, platelet CNFs (PL-CNFs), and irregular CNFs.

##### Regular CNFs

Regular CNF is a common carbon product in plastic carbonization (Fig. 3e). Gong et al. (Gong et al. 2015b; Gong et al. 2013b) found that the formation of CNFs was closely related to the shape and size of the catalysts. When nanosized catalysts coalesced and reconstructed into irregular polyhedral or round-like shapes during the carbonization process, graphitic layers will grow in different directions because the graphitic basal planes are parallel to the catalyst facets. Consequently, a solid core will form in CNFs.

##### Platelet CNFs (PL-CNFs)

PL-CNFs have a special structure; so that their graphitic layers are perpendicular to their growth axis (Fig. 3f). The catalysts in PL-CNFs are irregular polyhedral catalysts with hundreds of nanometers in diameter, which are relatively larger than the catalysts for the synthesis of other types of carbon filaments. The large size should be ascribed to the coalescence and sintering of catalysts particles (Gong et al. 2015b). The inner graphitic layer spacing of PL-CNFs is 0.34–0.36 nm, slightly larger than other carbons (0.34 nm). Furthermore, the graphitic layers of PL-CNFs are not continuous and have many defects, resulting in a low graphitization degree.

##### Irregular CNFs

It has been widely reported that abundant irregular CNFs were produced during CNTs synthesis from plastics (Cai et al. 2021; Veksha et al. 2022; Xu et al. 2021b; Yao et al. 2017). The appearance of irregular CNFs is caused by the agglomeration of catalyst nanoparticles and the piling up of mismatched graphene at edge sides due to stress energy (Haggar et al. 2022). The accumulation of catalyst nanoparticles will reduce accessible active sites for carbon growth. As a result, the graphene layers grown on the uncovered catalysts will stack up and exhibit irregular morphology. In general, reducing the particle size and preventing catalyst agglomeration are potential strategies to avoid the formation of irregular CNFs.

Compared with other carbon morphologies, carbon filaments are the most reported high-value carbon products from plastics. Numerous factors can affect the formation of carbon filaments, such as the intrinsic property and shape of catalysts, pyrolysis temperature, feedstock ratio, fabrication method, halogen radical, atmosphere, and pressure (Table 2). Increasing the proportion of light hydrocarbon intermediates is conducive to obtaining high-quality carbon filaments, and the controlled synthesis can be achieved by introducing catalysts and halogen radicals, choosing suitable plastics and fabrication methods, raising the ratio of plastics to catalysts, and increasing treatment temperature and time. However, it is still challenging to obtain uniform CNTs. One-pot and two-stage synthesis produce a mixture of carbon products, which will hamper the broad applications of CNTs, so further studies are required to improve the purity and quality of CNT products.

### Graphene

Top-down methods, including physical or chemical exfoliation, have been used to produce graphene from graphite. However, these methods suffer from either low yields or inferior qualities. Bottom-up approaches such as CVD using metal catalysts can produce high-quality graphene, but the carbon sources are expensive and explosive gaseous precursors. Thus plastic is a safe and economical option for graphene production (Kwon et al. 2019).

**Fig. 4:**
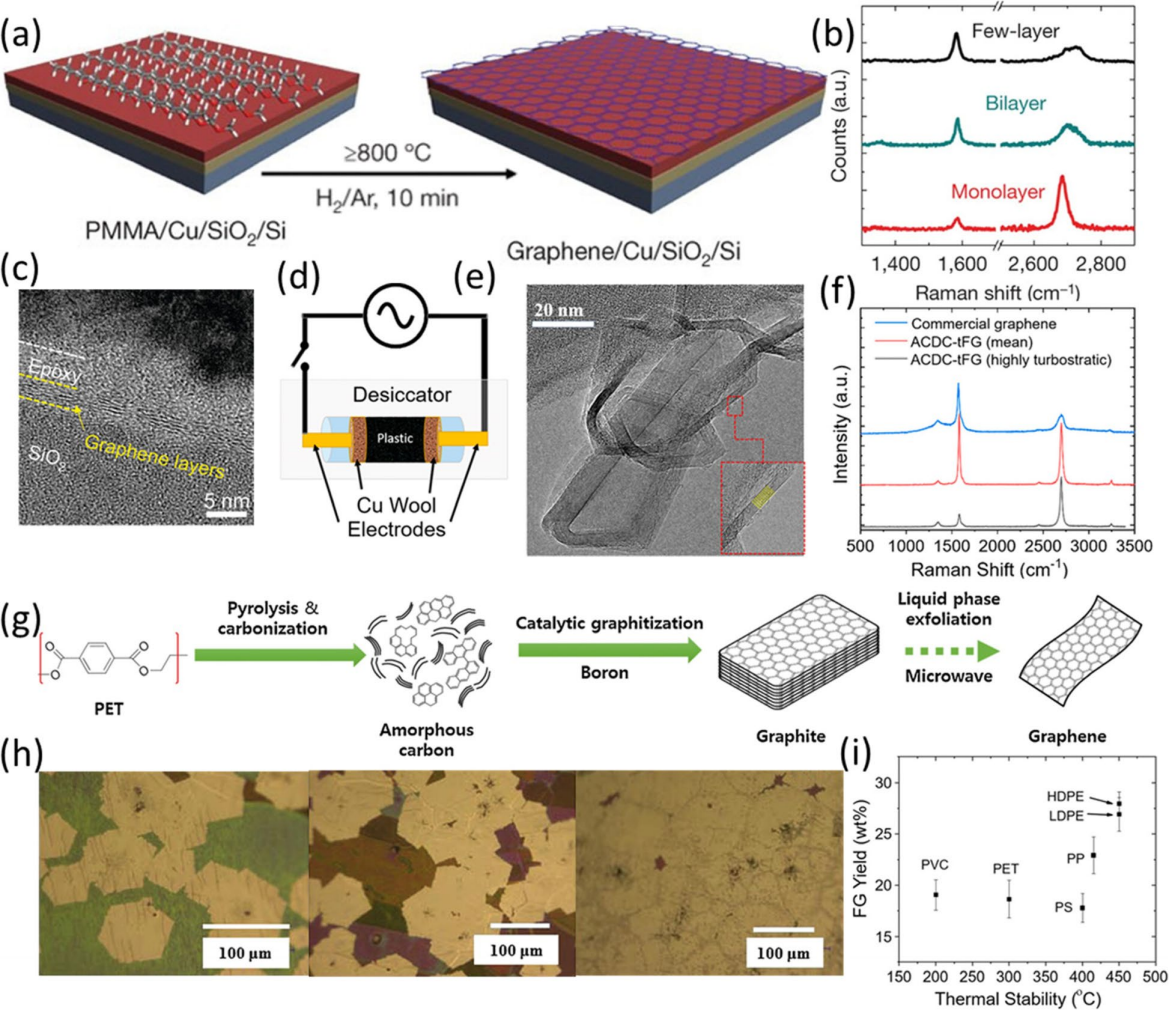
**a** Illustration of graphene synthesis by PMMA/Cu/SiO_2_/Si; **b** Raman spectra of monolayer, bilayer, and multilayer of graphene, reproduced with permission from Sun et al. (Sun et al. 2010), copyright 2010 Nature Springer; **c** HRTEM image of graphene formed on SiO_2_/Si substrate, reproduced with permission from Byun et al. (Byun et al. 2011), copyright 2011 American Chemical Society; **d** Schematic of the device for FJH method; **e** TEM image of flash graphene from HDPE; **f** Comparisons of Raman spectra after FJH treatment; **g** Schematic illustration of the preparation of graphite and graphene from PET, reproduced with permission from Ko et al. (Ko et al. 2020), copyright 2020 Elsevier; **h** Optical microscopic image of graphene crystals and the formation process of continuous graphene film, reproduced with permission from Sharma et al. (Sharma et al. 2014), copyright 2014 Elsevier; **i** The yields of flash graphene from different types of plastics, reproduced with permission from Algozeeb et al. (Algozeeb et al. 2020), copyright 2020 American Chemical Society

Several methods have been used to produce graphene with plastics, including CVD (Byun et al. 2011; Cui et al. 2017; Ruan et al. 2011; Sharma et al. 2014; Sun et al. 2010; Takami et al. 2014; Wang et al. 2012), flash Joule heating (FJH) (Algozeeb et al. 2020; Wyss et al. 2021), and pyrolysis and graphitization (Ko et al. 2020; Mensah et al. 2022). CVD has successfully transformed different types of plastics into graphene. Firstly, plastics are spin-coated or placed on metal foil catalysts to form a two-layer structure with/without the substrate of SiO_2_/Si or sapphire (Fig. 4a). Then, the composite is annealed at 800–1000 °C with a reductive gas flow (H_2_/Ar) in a low vacuum environment for a few minutes and high-quality graphene can be obtained (Ruan et al. 2011; Sun et al. 2010). The thickness of the product can be controlled to have a monolayer, bilayer, and a few layers by tuning the flow rate of H_2_ (Fig. 4b) (Sharma et al. 2014; Sun et al. 2010). H_2_ acts as a reducing agent and a carrier gas to remove carbon from plastic decomposition, and thus the lower the H_2_ flow, the more carbon source for the growth of multilayer graphene. Another sandwich structure was raised by Byun et al. (Byun et al. 2011) that the polymer layer was in the middle of a metal capping layer and SiO_2_/Si substrate. The metal capping layer can prevent the vaporization of decomposed molecules and catalyze graphene growth (Byun et al. 2011). However, only multilayer products were obtained, and the layer number cannot be controlled under this structure (Fig. 4c). Instead of forming composites prior to graphene synthesis, simply putting plastics at the upstream side of metal foil can also synthesize graphene. Interestingly, in this system, high-quality hexagonal graphene was obtained via lowering the gas injection rate and pre-annealing polycrystalline Cu foil (Sharma et al. 2014).

Recently, FJH with a sequent alternating current (AC) and direct current (DC) flash can convert mixed plastic waste into flash graphene in seconds. With 5 wt% carbon black as a catalyst, AC-FJH can reach ~ 2900 K, breaking C-C bonds, rearranging the carbon to graphene, and removing non-carbon volatiles. The subsequent DC-FJH can reach ~ 3100 K, and heal defects and disorders to obtain high-quality graphene (Fig. 4d-f) (Algozeeb et al. 2020). This process cannot control the layer of graphene and the product usually has multilayers, but the carbon yield from FJH can reach 20–30 wt% (Algozeeb et al. 2020), which is higher than the CVD method. FJH can also produce graphene from the pyrolysis ash of plastic waste (Wyss et al. 2021). Ko et al. (Ko et al. 2020) reported the method of pyrolysis and graphitization. In this process, PET was first converted into amorphous carbon at 900 °C, and then the carbon was graphitized by boron at 2400 °C. At last, the graphite was exfoliated to obtain graphene (Fig. 4g).

Cu and Ni foils are the mostly used metal catalysts in plastic conversion by CVD. Compared with Ni, Cu has a low solubility of carbon sources, so it can synthesize monolayer graphene and control layers in different gas environments (Fig. 4h) (Sun et al. 2010). Unlike Cu, Ni can reverse graphene to hydrocarbon products, which cuts graphene along certain directions (Sun et al. 2010). Also, Ni has stronger chemisorption of graphene and is more preferable to form graphene compared with Cu because carbon is more soluble in Ni (Byun et al. 2011). The thickness of metal catalysts can also make a difference in graphene quality. When the polymer is under a Ni catalyst, graphene grows by carbon diffusion into the catalyst and subsequent precipitation on the metal surface upon cooling. Therefore, too thin or too thick layers of the catalyst will hamper the synthesis process and result in inferior quality of graphene (Byun et al. 2011).

The impact of polymer types on product quality is minimal in the system using the Polymer/Cu/SiO_2_/Si composite as precursors. This is because carbon has a higher affinity on metal catalysts than heteroatoms, so carbon self-healing and atom rearrangement occurred to form high-quality graphene, resulting in a similar *I*_*D*_/*I*_*G*_ ratio in Raman spectra. Except for poly(methyl methacrylate) (PMMA), the higher oxygen content in PMMA will introduce more defects to the graphene (Cui et al. 2017). Among various polymers, PE, PP, and PS tend to have higher graphene yields due to their higher decomposition temperatures, which will lower the carbon supply rate. Thus, the generated carbon species will have more time to react with metal catalysts, resulting in higher carbon yields (Cui et al. 2017). Similar to CVD process, the carbon yield in FJH process is correlated with the thermal stability of plastics. The more stable the plastics, the higher the yields (Fig. 4i). For example, PE and PP produce more graphene than PET and PVC (Algozeeb et al. 2020). The thickness of polymer also affects the formation of graphene in CVD. The plastic close to the metal foil tends to form graphene with fewer defects, while the plastic adjacent to the substrate turns amorphous. Therefore, it was recommended to have a thinner polymer film in the first place (Byun et al. 2011).

Reaction temperature also impacts graphene quality. Low-temperature CVD results in a lower quality product or even amorphous carbon (Byun et al. 2011). For example, Sun et al. (Sun et al. 2010) discovered that at 800 °C, the *I*_*D*_/*I*_*G*_ peak ratio from Raman results was lower than 0.1, while that produced at 750 °C was about 0.35. After the reaction, lowering the cooling rate is beneficial to the reconstruction of hexagonal structure in graphene as it allows carbon to diffuse back to the metal surface and have a structural relaxation (Wang et al. 2012).

Studies have also been conducted to dope nitrogen for improvements of carbon properties. Traditionally, nitrogen doping can be achieved by using a NH_3_ gas flow during graphene growth by CVD or during the annealing treatment of synthesized graphene. N-doped graphene can also be obtained by simply mixing melamine with a plastic precursor prior to spin-coating on the metal foil. When using plasma-assisted CVD, using a N_2_/H_2_ gas mixture to treat grown graphene also helps incorporate nitrogen (Wang et al. 2012).

To sum up, CVD and FJH are effective ways to transform different types of plastics into graphene. The CVD technique can control the layers of graphene by tuning the H_2_/Ar gas flow, while FJH cannot. But FJH can achieve a higher carbon conversion rate than CVD. To obtain high-quality graphene in CVD, a moderate thickness of metal foil, plastics with high decomposition temperature, thin plastic film, and low cooling rate are encouraged. For FJH, using higher thermal stable plastic is beneficial to a higher yield of graphene. In the future, a further increase in the graphene yield with controllable layer structure in both systems will increase the feasibility of scale-up graphene production from waste carbon sources.

### Carbon nanosheets (CNS)

CNS is a 2D material of stacked graphene with few-nanometer thickness. CNS has been widely used in environmental remediation and energy recovery. However, the current methods to produce CNS are solid-state dichlorination, pyrolysis, and CVD. These methods require organic solvents, expensive templates or precursors, a sophisticated synthesis procedure, or a high vacuum environment (Wen et al. 2019). Thus, converting waste plastics to CNS via a facile approach is highly desirable. However, the conversion of plastics to CNS received less research attention. Carbonization parameters, including reactor design, catalyst type, and temperature are limited to a certain range.

There are mainly two reactors for the reaction: the quartz tube reactor and sealed reactor. The quartz tube is the mostly used reactor due to its advantages of easy operation and safety. Plastics are firstly mechanically or melted mixed with catalysts and then experience carbonization at 700–900 °C for up to three hours under an inert gas such as N_2_ and Ar. However, carbon volatiles may escape along with the gas before carbonization, resulting in carbon loss. Thus, a sealed autoclave reactor was developed to promote secondary reaction and increase carbon conversion and yields. For example, carbonizing PS plastics with MgO will produce monoaromatics and diaromatics in the first 30 minutes, which subsequently transformed into polycyclic aromatic hydrocarbons (PAHs) to form graphene layers (Ma et al. 2018). However, current studies did not discuss the influence and mechanisms of a pressurized environment.

**Fig. 5:**
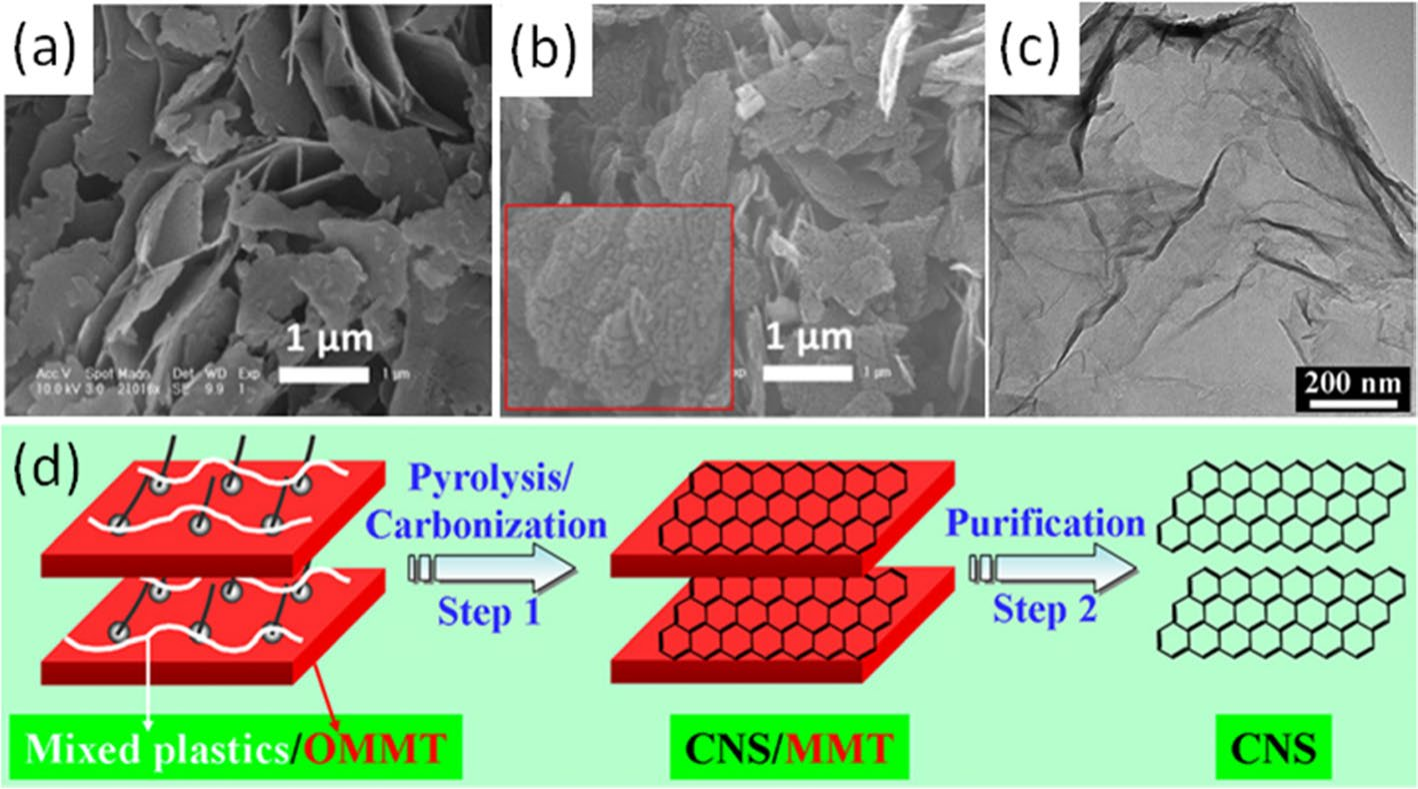
SEM image of **a** MgO nanosheet and **b** corresponding formed CNS, reproduced with permission from Ma et al. (Ma et al. 2020a), copyright 2020 IOP Publishing; **c** TEM image of CNS synthesized by mixed plastics/OMMT composite, reproduced with permission from Gong et al. (Gong et al. 2015a), copyright 2015 The Royal Society of Chemistry; **d** Scheme of CNS formation from mixed plastics/OMMT composite, reproduced with permission from Gong et al. (Gong et al. 2014d), copyright 2014 American Chemical Society

The production and yield of carbon nanosheets are dependent on the types of intermediates generated from the reaction between plastics and catalysts. There are mainly two catalysts used for CNS generation, MgO and OMMT. Both the catalysts serve as the template and catalyst spontaneously, and the weight ratios of plastics to catalyst are usually in the range of 1:1–1:6. MgO is more commonly used because it can be removed simply by washing with non-corrosive acids (Ma et al. 2018). The presence of MgO can bring several benefits to the carbonization system. Wen et al. (Wen et al. 2015) performed the carbonization of physically mixed PS/MgO in a quartz tube at 700 °C for six minutes. During the degradation, MgO promoted the transformation of C_2_-C_5_ to H_2_ and CH_4_, and further decomposed CH_4_ into H_2_ and carbon. In the meantime, MgO promotes PS degradation to aromatics with three or more rings with the sacrifice of diaromatics. Subsequently, the generated PAHs adsorb and assemble on MgO surface to form CNS. The morphology of obtained CNS is similar to that of MgO template (Fig. 5a and b). However, the low acid resistance of MgO makes it unreliable when degrading PVC due to the generation of HCl. Similarly, Mg(OH)_2_ sheets were also used as a catalyst and template for CNS formation (Wang et al. 2018). During heat treatments, Mg(OH)_2_ transforms into MgO and then performs carbonization.

OMMT is composed of stacked layers of two silica tetrahedrons fused to an edge-shared octahedral sheet of alumina, which can degrade plastics to light hydrocarbons and aromatics and further packing the intermediates into CNS (Fig. 5c and d) (Gong et al. 2014d). Compared with MgO, the alkylammonium deposited on OMMT introduces more Brønsted acid sites to catalyze the degradation of plastics (Wen et al. 2020), and the presence of OMMT can capture the PAHs from escape to increase the yield of CNS (Hong et al. 2012). However, the removal of OMMT requires hydrofluoric acid, which is hazardous and time-consuming (Ma et al. 2018). Unlike MgO, OMMT only acts as a template for nanosheet formation and cannot create pores on CNS. In addition, transition metals have been modified with these catalysts for promoted plastic transformation. Hong et al. (Hong et al. 2012) combined Fe and organophilic montmorillonite (OMT) as catalysts to transform PS to CNS. Dehydrogenation of aromatics occurred on Fe surface to form hexagonal rings, which could be transformed to CNS via epitaxial growth. OMT was responsible for constraining pyrolytic aromatics to facilitate graphene formation on the composites.

Aromatics compounds are reported to be the primary carbon source of graphitic nanosheets because of the favorable molecular structures of hexagonal rings (Hong et al. 2012). Experiments using different aromatics as carbon sources showed that PAHs, especially acenes, generate more carbon products compared with monoaromatics and diaromatics. Thus PS is prone to producing CNS under the same condition due to its higher proportion of aromatic compounds (Ma et al. 2018). Other plastics without aromatic rings can also produce carbon materials because the obtained liquid aliphatic compounds can transform into aromatics via a series of isomerization, dehydrogenation, and aromatization reactions, which can be utilized to generate graphitic sheets. However, their carbon yields are lower than PS. PVC has the lowest carbon yield because the generated HCl will deteriorate catalysts. PET has inferior carbonaceous products for two reasons. Firstly, PET mainly degrades to acids, alcohols and benzenes which are more difficult to be carbonized into CNS compared with PAHs. Also, oxygen from PET can oxidize the carbon product and reduce its quality (Ma et al. 2018; Wen et al. 2020).

To further improve the properties of CNS for broader applications, pores are introduced to increase the specific surface area. In addition to MgO template that can create a certain amount of mesopores below 50 nm (Wen et al. 2019; Wen et al. 2015), KOH activation is the most reported method for the formation of micropores and mesopores below 10 nm, making CNS a good candidate for pollutant adsorption (Gong et al. 2015a). Wang et al. (Wang et al. 2018) synthesized a composite of Mg(OH)_2_@CoZn-ZIFs to further increase porosity and functionalize the material. The evaporation of Zn species at high temperature can generate more micro/mesopores, while the nitrogen species in ZIFs will be introduced to the carbon product, further expanding the versatility of CNS.

In general, PAHs are the primary carbon sources for the growth of CNS. Thus, compared with other plastics, PS is the ideal candidate to prepare CNS due to the richness of aromatics in the structure. Using a sealed autoclave can potentially increase the yields of CNS because it can restrain aromatics from escaping and promote favorable secondary reactions. MgO and OMMT are used as both templates and catalysts to increase porosity and enhance CNS yields. However, other types of catalysts have rarely been reported. Until now, CNS formation mechanisms under different conditions (e.g., varied temperature and reaction durations) are still unclear and need further investigation.

### Carbon sphere

**Fig. 6:**
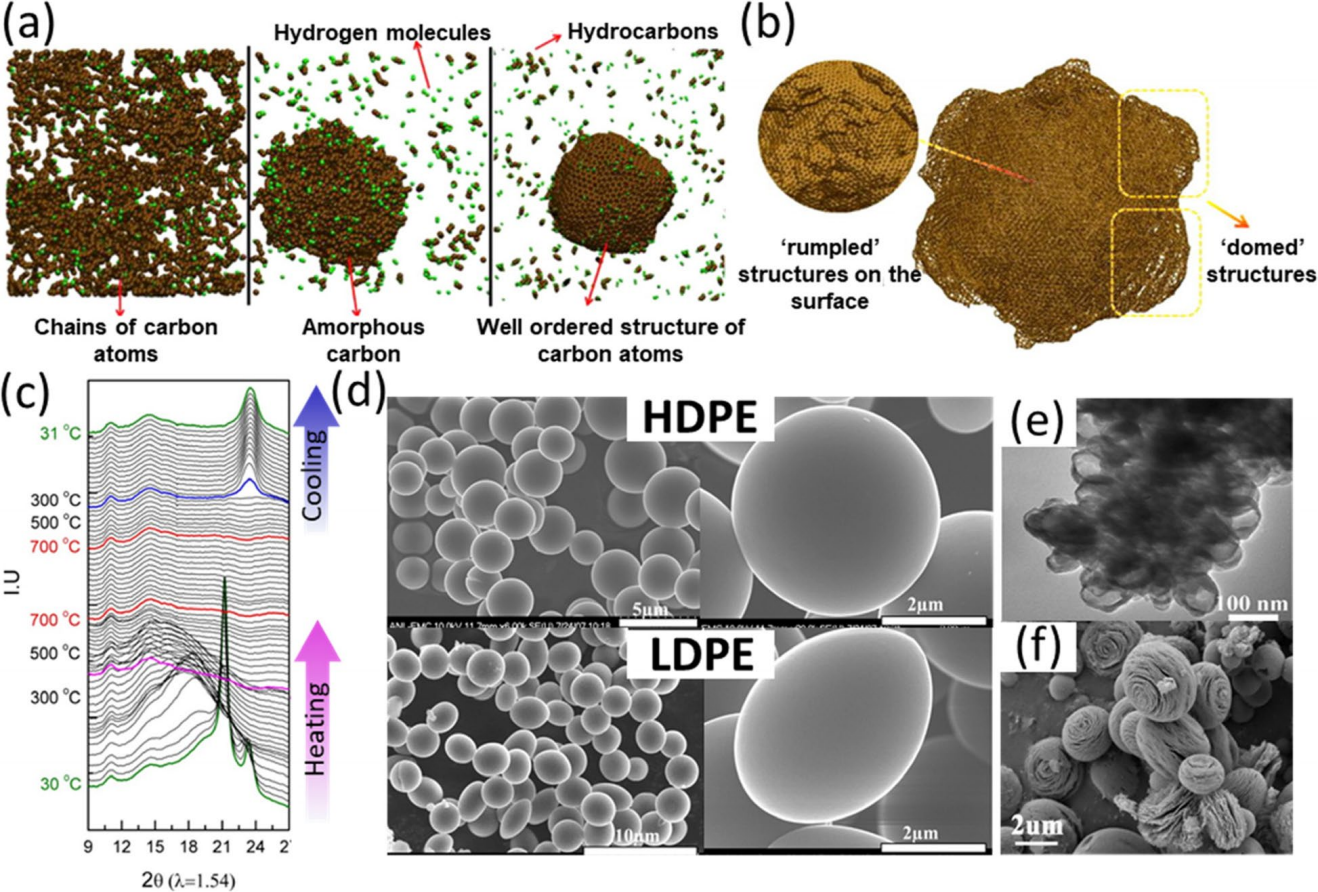
Molecular dynamics simulations of **a** carbon spheres formation and **b** rumpled and domed structure on the surface; **c** In-situ XRD spectra obtained during the transformation from PE to CS, reproduced with permission from Pol et al. (Pol et al. 2014), copyright 2014 American Chemical Society; **d** SEM images of CS obtained from HDPE and LDPE at 700 °C via autogenic reaction, reproduced with permission from Pol (Pol 2010), copyright 2010 American Chemical Society; **e** TEM image of aggregated hollow carbon spheres using OMMT/Co_3_O_4_ as template, reproduced with permission from Gong et al. (Gong et al. 2014b), copyright 2014 Elsevier; **f** SEM image of onion-like spheres obtain from PET-derived CS at 1500 °C under vacuum environment, reproduced with permission from Hu et al. (Hu et al. 2014), copyright 2014 American Chemical Society

Carbon spheres have been used as reinforcement for rubber and tyres, lubricants, printers, batteries, and paints. CS synthesized from plastics has solid core unless templates are used to produce hollow spheres. Currently, two processes have been applied in CS synthesis, including autogenic reaction (i.e. using an autoclave reactor) and atmospheric pressure reaction. For the autogenic reaction, a high pressure will be achieved in a closed autoclave along with the formation of small molecules decomposed from polymers. For example, PE breaks into short chains during the heating process and releases H_2_. The associated carbon proceeds polymerization to chains, and free hydrogen will form H_2_ or recombines with carbon compounds. During the cooling process, the high hydrogen content lowers the melting points of carbon-rich compounds, enabling the spheres to form monodispersed droplets (Pol et al. 2014). Thus, to keep surface energy minimum, spherical carbons are preferable during cooling (Fig. 6a-c) (Zhang et al. 2019b). Therefore, CS with a smooth surface was obtained. The sphere curvature is affected by the molten state before crystallization and the presence of multi-membered rings and defect structures (Pol et al. 2014). For an opened reactor, only CS with a rough surface and aggregated carbons were reported (Gong et al. 2013c; Gong et al. 2014b).

HDPE tends to produce spherical carbon in 3–5 μm in an autoclave after the treatments at 700 °C for 3 h, while LDPE yields semi-spherical carbon products under the same conditions (Fig. 6d). PS generates spherical carbon, but the sizes are diverse (4–10 μm) (Pol 2010). Sawant et al. (Sawant et al. 2013) observed a similar phenomenon: PP, HDPE, and LDPE produced spheres with smooth surfaces after 0 h retention time at 700 °C, while the products from PVC, PS, and PET had impurities of carbon particles on the surface. The particle impurities were ascribed to the intrinsic properties of charring polymers. The difference of the products from PS is contributed to the reaction time because a longer reaction time leads to a more complete reaction. For non-charring plastics, including PP and PE, oil containing aromatic hydrocarbons will form (< 500 °C), which will act as the precursors for sphere formation (> 500 °C) (Gong et al. 2013c; Sawant et al. 2013). However, for charring plastics, the backbone chain of charring plastics experiences cyclization, aromatization, or crosslinking instead of decomposition (< 500 °C), thus a carbon material frame will form (> 500 °C) and remain as irregular particles. To some charring polymers, such as chlorinated PVC, solutions have been raised to form monodispersed CS. By adding Cl-fixatives such as Fe_2_O_3_ and ZnCl_2_, the plastic can be transformed into carbon spheres instead of “sponge-like” carbon lump. This is because the Cl-fixatives accelerated dehydrochlorination and subsequent carbonization on the surface. This process occurred prior to polymer surface melting, preventing the spheres from binding and facilitating the generation of individual carbon spheres (Gong et al. 2013c; Zhou et al. 2022b). However, this strategy is only suitable for PVC. For PET, CS was formed under the atmosphere of pressurized CO_2_. CO_2_ can dissolve aromatic hydrocarbons from polymers, promoting the dissociation of plastic for CS formation (Wei et al. 2011).

Unlike other carbon morphologies, catalysts were less used in solid CS synthesis. The absence of a metal-based catalyst makes the product free from metal impurities and a purification process (Sawant et al. 2013). To produce hollow CS, Gong et al. (Gong et al. 2014a) used Co_3_O_4_ as a catalyst and template for sphere formation, but sole Co_3_O_4_ caused carbon agglomeration. Thus OMMT was combined with Co_3_O_4_ to help disperse Co_3_O_4_ and promote plastic degradation to light hydrocarbons for better CS growth (Fig. 6e) (Gong et al. 2014b). One of the advantages of a template is that the sphere size was controllable (from about 50 to over 100 nm), and the hollow structure increases the specific surface area of CS.

Reaction time and temperature are important factors in CS formation. Increasing reaction duration can improve the graphitization and yield of CS, meanwhile maintaining the shape and size (Pol 2010; Wei et al. 2011). At 650 °C, the CS yield increased from 13% to 47.5% with a prolonged retention time from 0 to 9 h. In terms of temperature, low reaction temperature of 600 °C resulted in agglomerated CS, but well-dispersed spheres were formed at 700 °C (Sawant et al. 2013). Carbonization at low temperature and short time leads to incomplete carbonization because of a low degree of aromatic condensation and the appearance of alkyl substitution, which shows irregular grains and flakes on the surface (Wei et al. 2011). Raising the temperature above 700 °C will intensify the carbonization degree by transforming disordered carbon into graphitic carbons (Pol 2010). Similarly, high-temperature post-treatment at 2400 °C can turn turbostratically disordered carbon into graphitic carbon, which promotes graphitization degree without changing carbon morphology (Pol and Thackeray 2011). However, if the temperature is too high (over 2800 °C), the surface will become rumpled due to the curving structure of longer-range crystallized graphite blocks (Pol et al. 2014). Interestingly, if the high-temperature treatment was performed in a vacuum furnace at 1500 °C, the smooth CS would transform into onion-like spheres with a rough surface and nanometer-scale flakes (Fig. 6f). However, only PET was investigated, and other types of plastics were not examined (Hu et al. 2014).

To sum up, smooth surface CS and aggregated rough particles can be obtained via autogenic reaction and atmospheric pressure reaction, respectively. To obtain high-quality CS with a smooth surface, we recommend increasing the reaction temperature (< 2800 °C), extending the reaction time, and using appropriate plastics. Non-charring plastics such as PP and PE are ideal feedstocks for CS synthesis, while charring plastics such as PET and PVC lead to irregular particles. The strategies of dechlorination and pressurization with CO_2_ could be used to promote CS formation from PVC and PET, respectively.

### Porous carbon

Due to its high specific surface area, low cost, adjustable pore structure, and good electrical conductivity, porous carbon has been widely applied in CO_2_ capture (Algozeeb et al. 2022; Song et al. 2022), solar steam evaporation (Zhang et al. 2019a), supercapacitor (Cheng et al. 2015; Ma et al. 2020b; Min et al. 2019; Zhang et al. 2021a), and battery (Min et al. 2020). Thus, the carbonization of waste plastics to porous carbons is beneficial for wide applications.

**Fig. 7:**
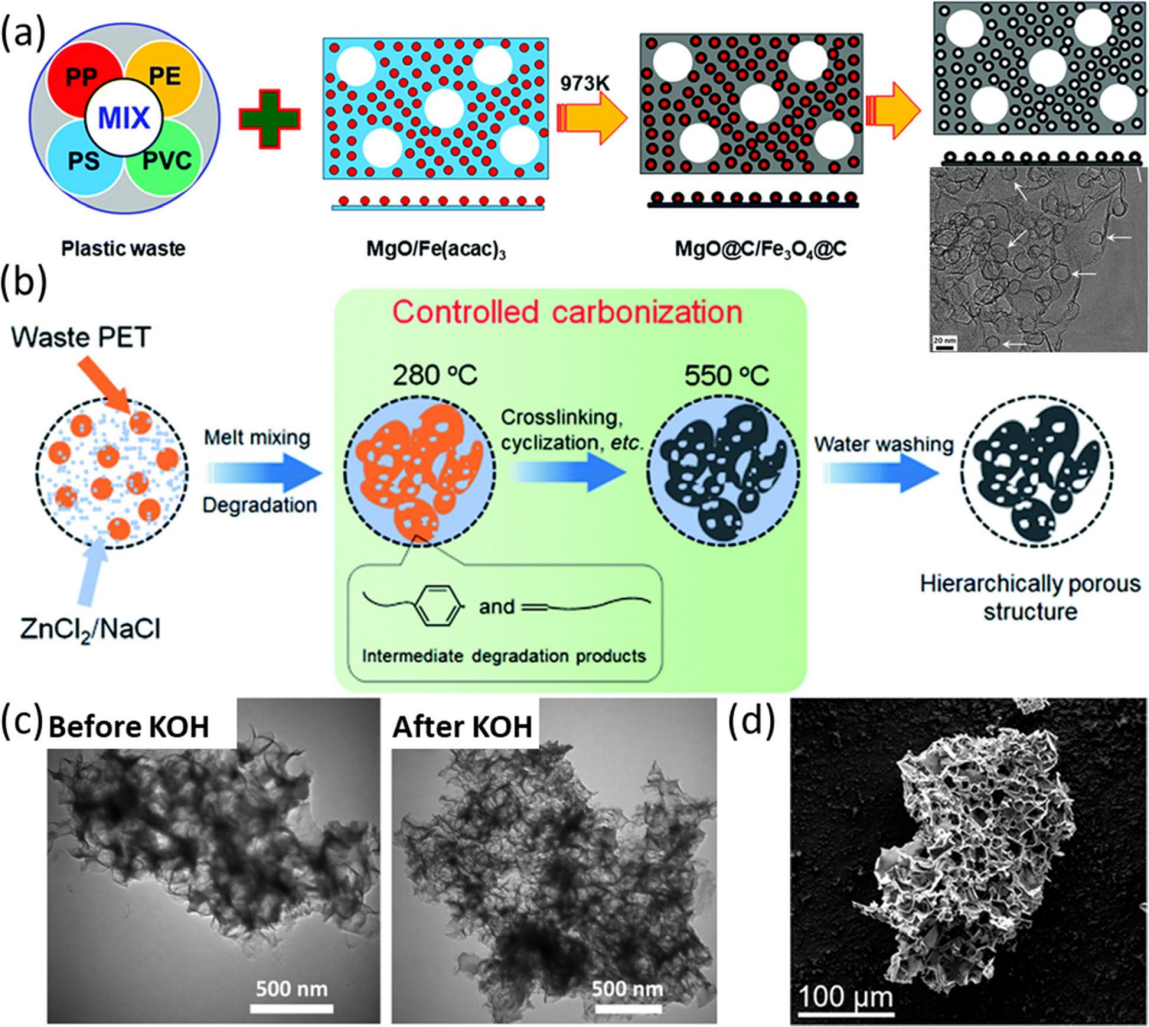
**a** Scheme of the formation of 3D porous hollow carbon spheres/porous carbon flakes, inset is the TEM image of HCl washed product, reproduced with permission from Min et al. (Min et al. 2020), copyright 2020 The Royal Society of Chemistry; **b** Scheme of PET carbonization using ZnCl_2_/NaCl eutectic salts, reproduced with permission from Zhang et al. (Zhang et al. 2019a), copyright 2019 The Royal Society of Chemistry; **c** TEM images of porous carbon synthesized before and after KOH treatment, reproduced with permission from Ma et al. (Ma et al. 2020b), copyright 2020 Elsevier; **d** SEM image of porous carbon synthesized with HDPE and KOAc, reproduced with permission from Algozeeb et al. (Algozeeb et al. 2022), copyright 2022 American Chemical Society

There are mainly two ways to introduce porous structure to plastic-derived carbons: pyrolysis with templates and activators-induced pore generation. Currently, the mostly commonly reported template is MgO due to its low price, facile fabrication, and controllable pore size and morphology. The facile removal of MgO by non-corrosive acids also makes it a favorable template. The evaporated small molecules from plastics can dehydrogenated and aromatized on MgO surface to form carbon materials that inherit the morphology of MgO. On this basis, Min et al., (Min et al. 2020) combined MgO with Fe (acac)_2_ as a combined template and transform different plastics (e.g. PE, PP, PS, and PVC) into a 3D hybrid containing hollow carbon spheres and porous carbon flakes. During heat treatments, Fe (acac)_2_ changed to Fe_3_O_4_ nanoparticles, which acted as a template for forming a hollow carbon sphere (Fig. 7a). Similarly, Mg(OH)_2_ was also used as a template because it would transform to MgO at high temperatures to produce cubic-shaped hollow carbon (Cheng et al. 2015). Other templates such as SBA-15 (Liang et al. 2019), silica (Zhang et al. 2018), and NaCl (Zhang et al. 2019a) were also used as a hard template in the pyrolysis of plastics for producing porous carbon. Salts can act as both a hard template and a catalyst to control the carbon yield and obtain hierarchically porous structures. Zhang et al. (Zhang et al. 2019a) used ZnCl_2_/NaCl eutectic salts to convert PET to porous carbons. A low amount of ZnCl_2_/NaCl promoted crosslinking of PET-decomposed products and improved carbon yield, and the eutectic salts acted as the templates for forming meso- and macro-pores (Fig. 7b).

During plastic carbonization, activation processes in in-situ pyrolysis or post-treatment will aid the formation of porous structures. Among various activators, KOH is mostly used to promote the formation of micropores and mesopores. KOH can facilitate the emission of more volatile compounds because carbon can react with KOH to form potassium carbonates (K_2_CO_3_), hydrogen, and potassium (Liew et al. 2019). When KOH is combined with metal oxide as macroporous templates, hierarchically porous carbon can be obtained (Fig. 7c). In addition, urea is beneficial to further developing pore structures because the released gases (CO_2_ and NH_3_) and introduced nitrogen will generate more defects in carbons, making it easier to interact with the KOH activator (Ma et al. 2020b). K_2_CO_3_ and K_2_O have a similar effect on pore formation (Ma et al. 2020b). However, the activation processes will introduce a large number of structural defects and oxygen-containing functional groups on the surface (Ma et al. 2020b). KOAc is another option as a carbon activator. Compared with KOH, KOAc is less corrosive, making it safer to use in scale-up productions (Algozeeb et al. 2022). Surface areas of products enlarged when the ratio of KOAc to plastics increased. The SEM image of porous carbon obtained with the KOAc/HDPE ratio of 4:1 is shown in Fig. 7d. However, at a higher ratio, the porous structures collapsed resulting in a lower surface area (Algozeeb et al. 2022). ZnCl_2_ is also used as an activator to treat PET-derived carbons. Compared with KOH in generating micropores and mesopores (0.4–4 nm), ZnCl_2_ only produces mesopores, but ZnCl_2_ activation can promote the graphitization degree of the carbonaceous product and reduce the content of surface functional groups (Zhang et al. 2021a). During activation processes, a high temperature usually leads to a larger surface area and helps decompose oxygen functionalities and decreases oxygen content. But over-heating may cause decomposition of carbon products due to etching by the metallic catalyst (e.g., potassium).

Among different plastics, charring plastics, including polyamide, PET, and PVC are more prone to producing porous carbons than non-charring plastics such as PE and PP (Algozeeb et al. 2022). This is because the pyrolysis of PP and PE mainly produces small molecules that are not favorable for carbon yield. PVC and PET do not decompose and instead experience aromatization, cross-linking, and cyclisation to form carbonaceous products (Yuwen et al., 2022). To overcome the low carbon yield of PP, microwave heating in concentrated sulfuric acid was proposed to induce sulfonation and oxidation reactions. The crosslinking of oxygen-containing and sulfonate groups helps increase the carbon yield and induce self-activation to etch and generate pores in subsequent pyrolysis (Yuwen et al., 2022). For PET, though it has higher carbon yields, the carbonization process is difficult to control because decomposition and crosslinking happen simultaneously. Adding salts such as ZnCl_2_ can help regulate the process (Zhang et al. 2019a).

In summary, pyrolysis with templates and pore-forming activators are two effective approaches to manufacturing porous carbons. Templates including MgO, Mg(OH)_2_, Fe (acac)_2_, SBA-15, silica, and NaCl can introduce meso- and macropores, while post-activation treatment by KOH and KOAc can produce micropores depending on the original carbon structures. Compared with non-charring plastics, PET, PVC, and polyamide are more favorable in porous carbon synthesis. One effective strategy to overcome the low carbon yields of PE and PP is to introduce functional groups (e.g., oxygen and sulfonate groups) and crosslink them to form graphitic carbons.

## Conclusions and perspectives

**Fig. 8:**
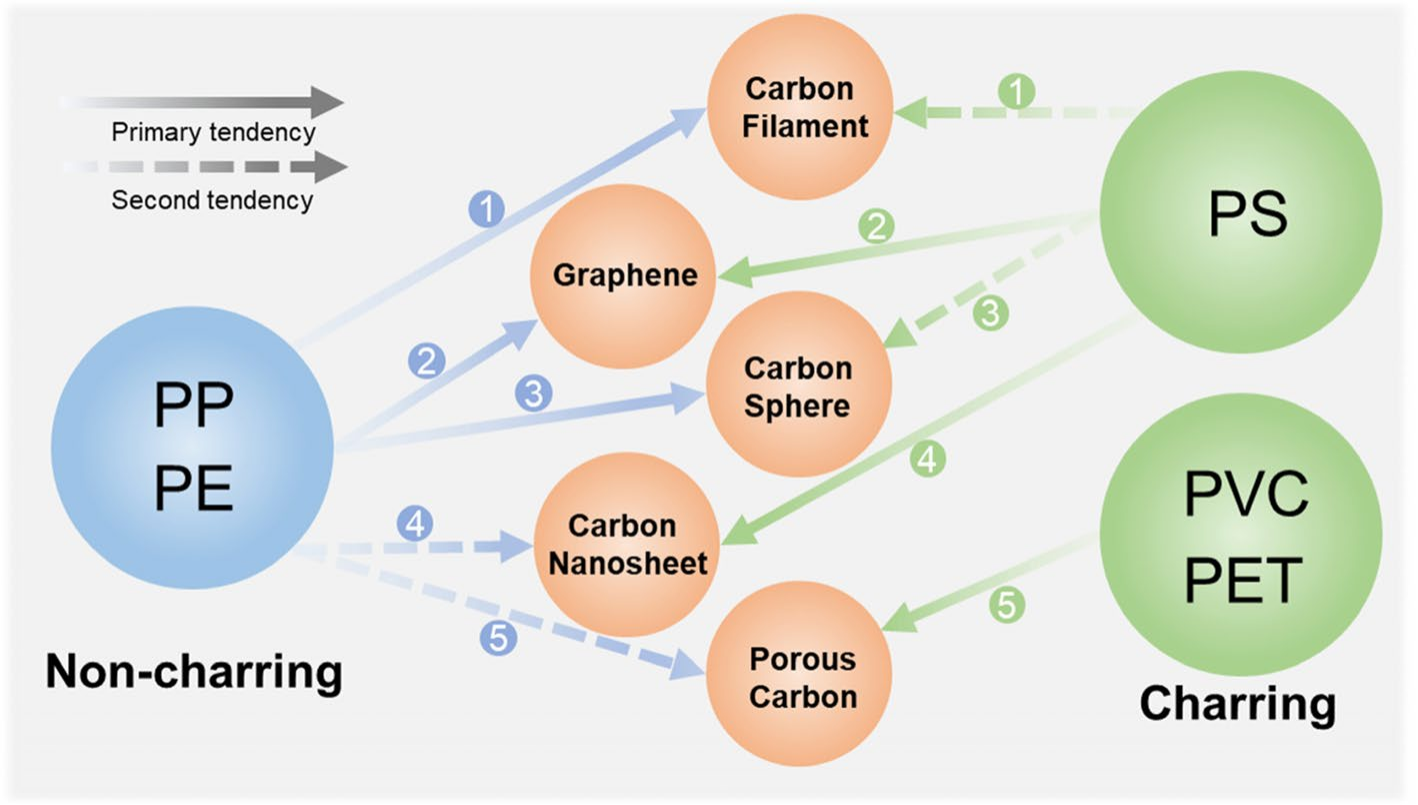
The carbonization tendency of different plastics. Number 1 arrow refers to refs (Aboul-Enein et al. 2018; Cai et al. 2021; Jie et al. 2020; Yao et al. 2022); Number 2 arrow refers to refs (Algozeeb et al. 2020; Cui et al. 2017; Ko et al. 2020); Number 3 arrow refers to refs (Gong et al. 2014b; Pol 2010; Sawant et al. 2013); Number 4 arrow refers to refs (Gong et al. 2015a; Ma et al. 2020a; Ma et al. 2018); Number 5 arrow refers to refs (Algozeeb et al. 2022; Ma et al. 2020b; Min et al. 2020)

As a carbon-rich feedstock, plastics have been used to produce various carbonaceous materials in different morphologies and structures and are appealing to economic CNM synthesis. Different types of plastics have their own carbonization features to yield distinct morphologies of CNMs (Fig. 8). Some plastics are less possible to evolve into specific morphologies due to their intrinsic structures, so efforts have been devoted to optimizing treatment conditions to resolve the limitations. This review has revealed the relationships between pyrolysis and catalysis processes by comparing and summarizing different studies of plastic carbonization. Identifying the intermediates produced from pyrolysis is the key to understanding the morphological evolution of the carbon products. Attention has been paid to the impacts of plastic types. It was discovered that non-charring plastics of PE and PP are prone to pyrolyzing into light hydrocarbons, which can act as the feedstock to form high-quality carbon filaments and carbon spheres. PS tends to decompose to aromatics, which are favorable to the formation of graphene and CNS. In terms of charring plastics of PET and PVC, their carbonization tends to form solid carbon material frames due to a series of reactions, including cyclization, aromatization, and crosslinking, so they are inclined to form porous carbon. This review also summarizes other impact factors such as catalysts, templates, temperature, pressure, and gaseous environment. Although different CNMs have been successfully synthesized from plastics, there are still challenges in improving CNMs quality and yields.

1. First, it remains challenging to produce uniform CNTs. The carbon filaments produced by one-pot methods are usually a mixture of CNTs, CNFs, and amorphous carbons, which limit the applications. The morphologies of carbon filaments produced by the two-stage treatment vary in wall number, diameter, and length. These problems are closely related to the shape, size, and nature of the metal catalysts. Thus, research endeavors will be more on the morphological control of existing catalysts and rational design of new, effective, and low-cost catalysts. The yield of carbon filaments produced by the two-stage method is usually below 20%, thereby the yield should also be substantially improved in future scale-up production.
2. Heteroatom doping is an effective way to alter the properties of CNMs, but only a few studies integrate it into plastic carbonization. In addition, CNMs produced from plastic carbonization are usually purified by acids because of the metal and oxide catalysts. Metals and acid in the waste solution should be recycled and reutilized. Reusable catalysts for plastic conversion should be exploited in future research.
3. There are still technical limitations on the formation of different morphological carbons. In terms of graphene synthesis, plastics with lower thermal stability still have low carbon yields. Among different plastics, PVC and PET are less investigated to synthesize graphene. For CNS, catalysts are limited to MgO and OMMT. Other types of catalysts are rarely reported. Modifications of MgO and OMMT are promising to introduce functionalities and fine-tune the properties of CNS, which are worthy of study in future research. Similarly, the effects of temperature, plastics/catalyst ratio, and reaction time are rarely discussed. Future research should conduct a more thorough investigation on precise control of CNS formation. CS, PVC and PET tend to produce irregular particles, which implies the limitation of autogenic reactions on mixed plastics conversion. Thus, techniques of adding Cl-fixatives and supercritical CO_2_ are required to convert PVC and PET, respectively. In the case of manufacturing porous carbon, non-charring plastics are not favorable due to the loss of light hydrocarbons, thus it is suggested that future research introduces functional groups prior to heat treatment to intensify the repolymerization and graphitization processes.
4. Each carbon morphology has a preference for a specific plastic feedstock. However, waste polymers are composed of various types of plastics and impurities. Current methods for mixed plastic conversion have limitations of low carbon yields. Thus, designing universal low-cost methods to convert different waste plastics is the key to realizing large-scale production and industrial applications. In addition, the effects of impurities such as metals, antioxidants, plasticizers, and fire retardants on the carbonization process are neglected in most research. These additives can enhance specific properties of plastics and may impact the carbon conversion processes, and thus should be investigated in future studies.

## Data Availability

No data will be shared.

## Abbreviations

PET: Polyethylene terephthalate
CNMs: Carbon nanomaterials
CNTs: Carbon nanotubes
CNS: Carbon nanosheets
CS: Carbon sphere
PE: Polyethylene
PP: Polypropylene
PS: Polystyrene
PVC: Polyvinyl chloride
TGA: Thermogravimetric analysis
HDPE: High-density polyethylene
LDPE: Low-density polyethylene
LLDPE: Linear low-density polyethylene
KOAc: Potassium acetate
OMMT: Organically modified montmorillonite
CVD: Chemical vapor deposition
CNFs: Carbon nanofibers
VLS: Vapor-liquid-solid
VSS: Vapor-solid-solid
NAAMs: Nanoporous anodic alumina membranes
PL-CNFs: Platelet carbon nanofibers
FJH: Flash Joule heating
AC: Alternating current
DC: Direct current
PMMA: Poly(methyl methacrylate)
PAHs: Polycyclic aromatic hydrocarbons
OMT: Organophilic montmorillonite

## References

[CR1] Aboul-Enein AA, Awadallah AE (2018). Production of nanostructured carbon materials using Fe–Mo/MgO catalysts via mild catalytic pyrolysis of polyethylene waste. Chem Eng J.

[CR2] Aboul-Enein AA, Awadallah AE, Abdel-Rahman AAH, Haggar AM (2018). Synthesis of multi-walled carbon nanotubes via pyrolysis of plastic waste using a two-stage process. Fuller Nanotub Carbon Nanostructures.

[CR3] Acomb JC, Wu C, Williams PT (2014). Control of steam input to the pyrolysis-gasification of waste plastics for improved production of hydrogen or carbon nanotubes. Appl Catal B Environ.

[CR4] Acomb JC, Wu C, Williams PT (2015). Effect of growth temperature and feedstock: catalyst ratio on the production of carbon nanotubes and hydrogen from the pyrolysis of waste plastics. J Anal Appl Pyrolysis.

[CR5] Acomb JC, Wu C, Williams PT (2016). The use of different metal catalysts for the simultaneous production of carbon nanotubes and hydrogen from pyrolysis of plastic feedstocks. Appl Catal B Environ.

[CR6] Adyel TM (2020). Accumulation of plastic waste during COVID-19. Science.

[CR7] Algozeeb WA, Savas PE, Luong DX, Chen W, Kittrell C, Bhat M, Shahsavari R, Tour JM (2020). Flash graphene from plastic waste. ACS Nano.

[CR8] Algozeeb WA, Savas PE, Yuan Z, Wang Z, Kittrell C, Hall JN, Chen W, Bollini P, Tour JM (2022). Plastic waste product captures carbon dioxide in nanometer pores. ACS Nano.

[CR9] Altalhi T, Kumeria T, Santos A, Losic D (2013). Synthesis of well-organised carbon nanotube membranes from non-degradable plastic bags with tuneable molecular transport: towards nanotechnological recycling. Carbon.

[CR10] Azara A, Belbessai S, Abatzoglou N (2022). A review of filamentous carbon nanomaterial synthesis via catalytic conversion of waste plastic pyrolysis products. J Environ Chem Eng.

[CR11] Baker RTK, Barber MA, Harris PS, Feates FS, Waite RJ (1972). Nucleation and growth of carbon deposits from the nickel catalyzed decomposition of acetylene. J Catal.

[CR12] Bazargan A, McKay G (2012). A review - synthesis of carbon nanotubes from plastic wastes. Chem Eng J.

[CR13] Borsodi N, Szentes A, Miskolczi N, Wu C, Liu X (2016). Carbon nanotubes synthetized from gaseous products of waste polymer pyrolysis and their application. J Anal Appl Pyrolysis.

[CR14] Byun SJ, Lim H, Shin GY, Han TH, Oh SH, Ahn JH, Choi HC, Lee TW (2011). Graphenes converted from polymers. J Phys Chem Lett.

[CR15] Cai N, Li X, Xia S, Sun L, Hu J, Bartocci P, Fantozzi F, Williams PT, Yang H, Chen H (2021). Pyrolysis-catalysis of different waste plastics over Fe/Al_2_O_3_ catalyst: high-value hydrogen, liquid fuels, carbon nanotubes and possible reaction mechanisms. Energy Convers Manag.

[CR16] Chao Y, Liu B, Rong Q, Zhang L, Guo S (2022). Porous carbon materials derived from discarded COVID-19 masks via microwave solvothermal method for lithium-sulfur batteries. Sci Total Environ.

[CR17] Cheng L, Zhang L, Chen X, Zhang Z (2015). Efficient conversion of waste polyvinyl chloride into nanoporous carbon incorporated with MnO_x_ exhibiting superior electrochemical performance for supercapacitor application. Electrochim Acta.

[CR18] Cho WS, Hamada E, Kondo Y, Takayanagi K (1996). Synthesis of carbon nanotubes from bulk polymer. Appl Phys Lett.

[CR19] Choi J, Yang I, Kim S, Cho SY, Lee S (2022). Upcycling plastic waste into high value-added carbonaceous materials. Macromol Rapid Commun.

[CR20] Cui L, Wang X, Chen N, Ji B, Qu L (2017). Trash to treasure: converting plastic waste into a useful graphene foil. Nanoscale.

[CR21] Deng J, You Y, Sahajwalla V, Joshi RK (2016). Transforming waste into carbon-based nanomaterials. Carbon.

[CR22] Elbaba IF, Williams PT (2012). Two stage pyrolysis-catalytic gasification of waste tyres: influence of process parameters. Appl Catal B Environ.

[CR23] Gangoli VS, Yick T, Bian F, Orbaek White A (2022). From waste plastics to carbon nanotube audio cables. C.

[CR24] Gong J, Chen X, Tang T (2019). Recent progress in controlled carbonization of (waste) polymers. Prog Polym Sci.

[CR25] Gong J, Feng J, Liu J, Muhammad R, Chen X, Jiang Z, Mijowska E, Wen X, Tang T (2013). Striking influence about HZSM-5 content and nickel catalyst on catalytic carbonization of polypropylene and polyethylene into carbon nanomaterials. Ind Eng Chem Res.

[CR26] Gong J, Liu J, Chen X, Jiang Z, Wen X, Mijowska E, Tang T (2014). One-pot synthesis of core/shell co@C spheres by catalytic carbonization of mixed plastics and their application in the photo-degradation of Congo red. J Mater Chem A.

[CR27] Gong J, Liu J, Chen X, Jiang Z, Wen X, Mijowska E, Tang T (2015). Converting real-world mixed waste plastics into porous carbon nanosheets with excellent performance in the adsorption of an organic dye from wastewater. J Mater Chem A.

[CR28] Gong J, Liu J, Jiang Z, Chen X, Wen X, Mijowska E, Tang T (2014). Converting mixed plastics into mesoporous hollow carbon spheres with controllable diameter. Appl Catal B Environ.

[CR29] Gong J, Liu J, Jiang Z, Chen X, Wen X, Mijowska E, Tang T (2015). New insights into the role of lattice oxygen in the catalytic carbonization of polypropylene into high value-added carbon nanomaterials. New J Chem.

[CR30] Gong J, Liu J, Jiang Z, Feng J, Chen X, Wang L, Mijowska E, Wen X, Tang T (2014). Striking influence of chain structure of polyethylene on the formation of cup-stacked carbon nanotubes/carbon nanofibers under the combined catalysis of CuBr and NiO. Appl Catal B Environ.

[CR31] Gong J, Liu J, Jiang Z, Wen X, Chen X, Mijowska E, Wang Y, Tang T (2013). Effect of the added amount of organically-modified montmorillonite on the catalytic carbonization of polypropylene into cup-stacked carbon nanotubes. Chem Eng J.

[CR32] Gong J, Liu J, Ma L, Wen X, Chen X, Wan D, Yu H, Jiang Z, Borowiak-Palen E, Tang T (2012). Effect of cl/Ni molar ratio on the catalytic conversion of polypropylene into cu-Ni/C composites and their application in catalyzing “click” reaction. Appl Catal B Environ.

[CR33] Gong J, Liu J, Wan D, Chen X, Wen X, Mijowska E, Jiang Z, Wang Y, Tang T (2012). Catalytic carbonization of polypropylene by the combined catalysis of activated carbon with Ni_2_O_3_ into carbon nanotubes and its mechanism. Appl Catal A Gen.

[CR34] Gong J, Michalkiewicz B, Chen X, Mijowska E, Liu J, Jiang Z, Wen X, Tang T (2014). Sustainable conversion of mixed plastics into porous carbon nanosheets with high performances in uptake of carbon dioxide and storage of hydrogen. ACS Sustain Chem Eng.

[CR35] Gong J, Yao K, Liu J, Jiang Z, Chen X, Wen X, Mijowska E, Tian N, Tang T (2013). Striking influence of Fe_2_O_3_ on the “catalytic carbonization” of chlorinated poly (vinyl chloride) into carbon microspheres with high performance in the photo-degradation of Congo red. J Mater Chem A.

[CR36] Gong J, Yao K, Liu J, Wen X, Chen X, Jiang Z, Mijowska E, Tang T (2013). Catalytic conversion of linear low density polyethylene into carbon nanomaterials under the combined catalysis of Ni_2_O_3_ and poly (vinyl chloride). Chem Eng J.

[CR37] Ha S, Hyun JC, Kwak JH, Lim H-D, Youn BS, Cho S, Jin H-J, Lim H-K, Lee SM, Yun YS (2022). Waste-induced pyrolytic carbon nanotube forest as a catalytic host electrode for high-performance aluminum metal anodes. Chem Eng J.

[CR38] Haggar AM, Awadallah AE, Aboul-Enein AA, Sayed GH (2022). Non-oxidative conversion of real low density polyethylene waste into hydrogen and carbon nanomaterials over MgO supported bimetallic co-Mo catalysts with different total co-Mo contents. Chem Eng Sci.

[CR39] Harussani MM, Sapuan SM, Rashid U, Khalina A, Ilyas RA (2022). Pyrolysis of polypropylene plastic waste into carbonaceous char: priority of plastic waste management amidst COVID-19 pandemic. Sci Total Environ.

[CR40] Hong N, Wang B, Song L, Hu S, Tang G, Wu Y, Hu Y (2012). Low-cost, facile synthesis of carbon nanosheets by thermal pyrolysis of polystyrene composite. Mater Lett.

[CR41] Hu H, Gao L, Chen C, Chen Q (2014). Low-cost, acid/alkaline-resistant, and fluorine-free superhydrophobic fabric coating from onionlike carbon microspheres converted from waste polyethylene terephthalate. Environ Sci Technol.

[CR42] Hu K, Zhou P, Yang Y, Hall T, Nie G, Yao Y, Duan X, Wang S (2022). Degradation of microplastics by a thermal Fenton reaction. ACS ES T Eng.

[CR43] Huang Z, Zheng Y, Zhang H, Li F, Zeng Y, Jia Q, Zhang J, Li J, Zhang S (2021). High-yield production of carbon nanotubes from waste polyethylene and fabrication of graphene-carbon nanotube aerogels with excellent adsorption capacity. J Mater Sci Technol.

[CR44] Iijima S (1991). Helical microtubules of graphitic carbon. Nature.

[CR45] Jia J, Veksha A, Lim T-T, Lisak G (2022). Temperature-dependent synthesis of multi-walled carbon nanotubes and hydrogen from plastic waste over A-site-deficient perovskite La_0.8_Ni_1-x_Co_x_O_3-δ_. Chemosphere.

[CR46] Jiang K, Feng C, Liu K, Fan S (2007). A vapor-liquid-solid model for chemical vapor deposition growth of carbon nanotubes. J Nanosci Nanotechnol.

[CR47] Jie X, Li W, Slocombe D, Gao Y, Banerjee I, Gonzalez-Cortes S, Yao B, AlMegren H, Alshihri S, Dilworth J, Thomas J, Xiao T, Edwards P (2020). Microwave-initiated catalytic deconstruction of plastic waste into hydrogen and high-value carbons. Nat Catal.

[CR48] Kang J, Zhou L, Duan X, Sun H, Ao Z, Wang S (2019). Degradation of cosmetic microplastics via functionalized carbon nanosprings. Matter.

[CR49] Ko S, Kwon YJ, Lee JU, Jeon Y-P (2020). Preparation of synthetic graphite from waste PET plastic. J Ind Eng Chem.

[CR50] Kong Q, Zhang J (2007). Synthesis of straight and helical carbon nanotubes from catalytic pyrolysis of polyethylene. Polym Degrad Stab.

[CR51] Kwon S-J, Seo H-K, Ahn S, Lee T-W (2019). Value-added recycling of inexpensive carbon sources to graphene and carbon nanotubes. Adv Sustain Syst.

[CR52] Li W-J, Kuo J-H, Yang R-X, Wey M-Y (2019). Effect of preparation solvent and calcination atmosphere on Ni@SiO_2_ catalyst for simultaneous production of hydrogen and carbon nanotubes from simulated plastic waste syngas. Energy Technol.

[CR53] Liang K, Liu L, Wang W, Yu Y, Wang Y, Zhang L, Ma C, Chen A (2019). Conversion of waste plastic into ordered mesoporous carbon for electrochemical applications. J Mater Res.

[CR54] Liew R, Chai C, Yek P, Phang X, Chong M, Nam W, Su M, Lam W, Ma N, Lam S (2019). Innovative production of highly porous carbon for industrial effluent remediation via microwave vacuum pyrolysis plus sodium-potassium hydroxide mixture activation. J Clean Prod.

[CR55] Liu J, Jiang Z, Yu H, Tang T (2011). Catalytic pyrolysis of polypropylene to synthesize carbon nanotubes and hydrogen through a two-stage process. Polym Degrad Stab.

[CR56] Lu H, Diaz DJ, Czarnecki NJ, Zhu C, Kim W, Shroff R, Acosta DJ, Alexander BR, Cole HO, Zhang Y, Lynd NA, Ellington AD, Alper HS (2022). Machine learning-aided engineering of hydrolases for PET depolymerization. Nature.

[CR57] Ma C, Liu X, Min J, Li J, Gong J, Wen X, Chen X, Tang T, Mijowska E (2020). Sustainable recycling of waste polystyrene into hierarchical porous carbon nanosheets with potential applications in supercapacitors. Nanotechnology.

[CR58] Ma C, Min J, Gong J, Liu X, Mu X, Chen X, Tang T (2020). Transforming polystyrene waste into 3D hierarchically porous carbon for high-performance supercapacitors. Chemosphere.

[CR59] Ma J, Liu J, Song J, Tang T (2018). Pressurized carbonization of mixed plastics into porous carbon sheets on magnesium oxide. RSC Adv.

[CR60] Mensah K, Mahmoud H, Fujii M, Shokry H (2022). Novel nano-ferromagnetic activated graphene adsorbent extracted from waste for dye decolonization. J Water Process Eng.

[CR61] Min J, Wen X, Tang T, Chen X, Huo K, Gong J, Azadmanjiri J, He C, Mijowska E (2020). A general approach towards carbonization of plastic waste into a well-designed 3D porous carbon framework for super lithium-ion batteries. Chem Commun.

[CR62] Min J, Zhang S, Li J, Klingeler R, Wen X, Chen X, Zhao X, Tang T, Mijowska E (2019). From polystyrene waste to porous carbon flake and potential application in supercapacitor. Waste Manag.

[CR63] Modekwe HU, Mamo MA, Moothi K, Daramola MO (2021). Effect of different catalyst supports on the quality, yield and morphology of carbon nanotubes produced from waste polypropylene plastics. Catalysts.

[CR64] Moisala A, Nasibulin AG, Kauppinen EI (2003). The role of metal nanoparticles in the catalytic production of single-walled carbon nanotubes—a review. J Phys Condens Matter.

[CR65] Orbaek White A, Hedayati A, Yick T, Gangoli VS, Niu Y, Lethbridge S, Tsampanakis I, Swan G, Pointeaux L, Crane A, Charles R, Sallah-Conteh J, Anderson AO, Davies ML, Palmer RE (2022). Synthesis of carbon nanotubes via liquid injection chemical vapour deposition as a vector for the chemical recycling of waste composite carbon sources. Nanomaterials.

[CR66] Panahi A, Wei Z, Song G, Levendis YA (2019). Influence of stainless-steel catalyst substrate type and pretreatment on growing carbon nanotubes from waste postconsumer plastics. Ind Eng Chem Res.

[CR67] PlasticsEurope, 2021. Plastics—the facts 2021 an analysis of european plastics production, demand and waste data

[CR68] Pol VG (2010). Upcycling: converting waste plastics into paramagnetic, conducting, solid, pure carbon microspheres. Environ Sci Technol.

[CR69] Pol VG, Thackeray MM (2011). Spherical carbon particles and carbon nanotubes prepared by autogenic reactions: evaluation as anodes in lithium electrochemical cells. Energy Environ Sci.

[CR70] Pol VG, Wen J, Lau KC, Callear S, Bowron DT, Lin CK, Deshmukh SA, Sankaranarayanan S, Curtiss LA, David WIF, Miller DJ, Thackeray MM (2014). Probing the evolution and morphology of hard carbon spheres. Carbon.

[CR71] Ribeiro RS, Vieira O, Fernandes R, Roman FF, Diaz de Tuesta JL, Silva AMT, Gomes HT (2022). Synthesis of low-density polyethylene derived carbon nanotubes for activation of persulfate and degradation of water organic micropollutants in continuous mode. J Environ Manag.

[CR72] Ruan G, Sun Z, Peng Z, Tour JM (2011). Growth of graphene from food, insects, and waste. ACS Nano.

[CR73] Sawant SY, Somani RS, Panda AB, Bajaj HC (2013). Utilization of plastic wastes for synthesis of carbon microspheres and their use as a template for nanocrystalline copper (II) oxide hollow spheres. ACS Sustain Chem Eng.

[CR74] Shah K, Patel S, Halder P, Kundu S, Marzbali MH, Hakeem IG, Pramanik BK, Chiang K, Patel T (2022). Conversion of pyrolytic non-condensable gases from polypropylene co-polymer into bamboo-type carbon nanotubes and high-quality oil using biochar as catalyst. J Environ Manag.

[CR75] Sharma S, Kalita G, Hirano R, Shinde SM, Papon R, Ohtani H, Tanemura M (2014). Synthesis of graphene crystals from solid waste plastic by chemical vapor deposition. Carbon.

[CR76] Song C, Zhang B, Hao L, Min J, Liu N, Niu R, Gong J, Tang T (2022). Converting poly (ethylene terephthalate) waste into N-doped porous carbon as CO_2_ adsorbent and solar steam generator. Green Energy Environ.

[CR77] Song R, Jiang Z, Bi W, Cheng W, Lu J, Huang B, Tang T (2007). The combined catalytic action of solid acids with nickel for the transformation of polypropylene into carbon nanotubes by pyrolysis. Chem A Eur J.

[CR78] Sridhar V, Park H (2020). Transforming waste poly (ethylene terephthalate) into nitrogen doped carbon nanotubes and its utility in oxygen reduction reaction and bisphenol-a removal from contaminated water. Materials.

[CR79] Sun Z, Yan Z, Yao J, Beitler E, Zhu Y, Tour JM (2010). Growth of graphene from solid carbon sources. Nature.

[CR80] Takami T, Seino R, Yamazaki K, Ogino T (2014). Graphene film formation on insulating substrates using polymer films as carbon source. J Phys D Appl Phys.

[CR81] Tessonnier JP, Su DS (2011). Recent progress on the growth mechanism of carbon nanotubes: a review. Chem Sus Chem.

[CR82] Thivasasith A, Rodaum C, Nunthakitgoson W, Assavapanumat S, Wattanakit C (2022). Fine-tuning the catalytic cracking-assisted synthesis of plastic-derived MWCNTs-supported metal oxides for methanol electrooxidation. Carbon Trends.

[CR83] Thompson RC, Swan SH, Moore CJ, Vom Saal FS (2009). Our plastic age. Philos Trans R Soc B Biol Sci.

[CR84] Tripathi P, Durbach S, Coville N (2017). Synthesis of multi-walled carbon nanotubes from plastic waste using a stainless-steel CVD reactor as catalyst. Nanomaterials.

[CR85] Veksha A, Bin Mohamed Amrad MZ, Chen WQ, Binte Mohamed DK, Tiwari SB, Lim T, Lisak G (2022). Tailoring Fe_2_O_3_–Al_2_O_3_ catalyst structure and activity via hydrothermal synthesis for carbon nanotubes and hydrogen production from polyolefin plastics. Chemosphere.

[CR86] Vieira O, Ribeiro RS, Diaz de Tuesta JL, Gomes HT, Silva AMT (2022). A systematic literature review on the conversion of plastic wastes into valuable 2D graphene-based materials. Chem Eng J.

[CR87] Vollmer I, Jenks MJF, Roelands MCP, White RJ, van Harmelen T, de Wild P, van der Laan GP, Meirer F, Keurentjes JTF, Weckhuysen BM (2020). Beyond mechanical recycling: giving new life to plastic waste. Angew Chem Int Ed.

[CR88] Wang CD, Yuen MF, Ng TW, Jha SK, Lu ZZ, Kwok SY, Wong TL, Yang X, Lee CS, Lee ST, Zhang WJ (2012). Plasma-assisted growth and nitrogen doping of graphene films. Appl Phys Lett.

[CR89] Wang G, Liu L, Zhang L, Fu X, Liu M, Zhang Y, Yu Y, Chen A (2018). Porous carbon nanosheets prepared from plastic wastes for supercapacitors. J Electron Mater.

[CR90] Wang J, Shen B, Lan M, Kang D, Wu C (2020). Carbon nanotubes (CNTs) production from catalytic pyrolysis of waste plastics: the influence of catalyst and reaction pressure. Catal Today.

[CR91] Wei L, Yan N, Chen Q (2011). Converting poly (ethylene terephthalate) waste into carbon microspheres in a supercritical CO_2_ system. Environ Sci Technol.

[CR92] Wen X, Chen X, Tian N, Gong J, Liu J, Rümmeli MH, Chu PK, Mijiwska E, Tang T (2014). Nanosized carbon black combined with Ni_2_O_3_ as “universal” catalysts for synergistically catalyzing carbonization of polyolefin wastes to synthesize carbon nanotubes and application for supercapacitors. Environ Sci Technol.

[CR93] Wen Y, Kierzek K, Chen X, Gong J, Liu J, Niu R, Mijowska E, Tang T (2019). Mass production of hierarchically porous carbon nanosheets by carbonizing “real-world” mixed waste plastics toward excellent-performance supercapacitors. Waste Manag.

[CR94] Wen Y, Kierzek K, Min J, Chen X, Gong J, Niu R, Wen X, Azadmanjiri J, Mijowska E, Tang T (2020). Porous carbon nanosheet with high surface area derived from waste poly (ethylene terephthalate) for supercapacitor applications. J Appl Polym Sci.

[CR95] Wen Y, Liu J, Song J, Gong J, Chen H, Tang T (2015). Conversion of polystyrene into porous carbon sheets and hollow carbon shells over different magnesium oxide templates for efficient removal of methylene blue. RSC Adv.

[CR96] Williams PT (2021). Hydrogen and carbon nanotubes from pyrolysis-catalysis of waste plastics: a review. Waste Biomass Valori.

[CR97] Wu C, Nahil MA, Miskolczi N, Huang J, Williams PT (2014). Processing real-world waste plastics by pyrolysis-reforming for hydrogen and high-value carbon nanotubes. Environ Sci Technol.

[CR98] Wu C, Williams PT (2009). Hydrogen production by steam gasification of polypropylene with various nickel catalysts. Appl Catal B Environ.

[CR99] Wulan PPDK, Sidauruk JOD, Ningtyas JA (2018). The effect of pyrolysis temperature and time of polypropylene on quality of carbon nanotube with flame synthesis method. E3S Web Conf.

[CR100] Wyss KM, Beckham JL, Chen W, Luong DX, Hundi P, Raghuraman S, Shahsavari R, Tour JM (2021). Converting plastic waste pyrolysis ash into flash graphene. Carbon.

[CR101] Xu D, Xiong Y, Ye J, Su Y, Dong Q, Zhang S (2020). Performances of syngas production and deposited coke regulation during co-gasification of biomass and plastic wastes over Ni/γ-Al_2_O_3_ catalyst: role of biomass to plastic ratio in feedstock. Chem Eng J.

[CR102] Xu D, Xiong Y, Zhang S, Su Y (2021). The synergistic mechanism between coke depositions and gas for H_2_ production from co-pyrolysis of biomass and plastic wastes via char supported catalyst. Waste Manag.

[CR103] Xu D, Yang S, Su Y, Shi L, Zhang S, Xiong Y (2021). Simultaneous production of aromatics-rich bio-oil and carbon nanomaterials from catalytic co-pyrolysis of biomass/plastic wastes and in-line catalytic upgrading of pyrolysis gas. Waste Manag.

[CR104] Xu D, Yang S, Su Y, Xiong Y, Zhang S (2021). Catalytic conversion of plastic wastes using cost-effective bauxite residue as catalyst into H_2_-rich syngas and magnetic nanocomposites for chrome (VI) detoxification. J Hazard Mater.

[CR105] Yang R, Chuang K, Wey M (2015). Effects of nickel species on Ni/Al_2_O_3_ catalysts in carbon nanotube and hydrogen production by waste plastic gasification: bench- and pilot-scale tests. Energy Fuel.

[CR106] Yang R, Chuang K, Wey M (2016). Carbon nanotube and hydrogen production from waste plastic gasification over Ni/Al-SBA-15 catalysts: effect of aluminum content. RSC Adv.

[CR107] Yang R, Chuang K, Wey M (2018). Effects of temperature and equivalence ratio on carbon nanotubes and hydrogen production from waste plastic gasification in fluidized bed. Energy Fuel.

[CR108] Yang R, Wu S, Chuang K, Wey M (2020). Co-production of carbon nanotubes and hydrogen from waste plastic gasification in a two-stage fluidized catalytic bed. Renew Energy.

[CR109] Yang Z, Zhang Q, Luo G, Huang J, Zhao M, Wei F (2010). Coupled process of plastics pyrolysis and chemical vapor deposition for controllable synthesis of vertically aligned carbon nanotube arrays. Appl Phys A Mater Sci Process.

[CR110] Yao D, Li H, Dai Y, Wang C-H (2021). Impact of temperature on the activity of Fe-Ni catalysts for pyrolysis and decomposition processing of plastic waste. Chem Eng J.

[CR111] Yao D, Li H, Mohan BC, Prabhakar AK, Dai Y, Wang CH (2022). Conversion of waste plastic packings to carbon nanomaterials: investigation into catalyst material, waste type, and product applications. ACS Sustain Chem Eng.

[CR112] Yao D, Wu C, Yang H, Zhang Y, Nahil MA, Chen Y, Williams PT, Chen H (2017). Co-production of hydrogen and carbon nanotubes from catalytic pyrolysis of waste plastics on Ni-Fe bimetallic catalyst. Energy Convers Manag.

[CR113] Yao D, Yang H, Chen H, Williams PT (2018). Investigation of nickel-impregnated zeolite catalysts for hydrogen/syngas production from the catalytic reforming of waste polyethylene. Appl Catal B Environ.

[CR114] Yao D, Yang H, Hu Q, Chen Y, Chen H, Williams PT (2021). Carbon nanotubes from post-consumer waste plastics: investigations into catalyst metal and support material characteristics. Appl Catal B Environ.

[CR115] Yao D, Zhang Y, Williams PT, Yang H, Chen H (2018). Co-production of hydrogen and carbon nanotubes from real-world waste plastics: influence of catalyst composition and operational parameters. Appl Catal B Environ.

[CR116] Yu H, Jiang Z, Gilman JW, Kashiwagi T, Liu J, Song R, Tang T (2009). Promoting carbonization of polypropylene during combustion through synergistic catalysis of a trace of halogenated compounds and Ni_2_O_3_ for improving flame retardancy. Polymer.

[CR117] Yu J, Sun L, Ma C, Qiao Y, Yao H (2016). Thermal degradation of PVC: a review. Waste Manag.

[CR118] Zhang B, Song C, Liu C, Min J, Azadmanjiri J, Ni Y, Niu R, Gong J, Zhao Q, Tang T (2019). Molten salts promoting the “controlled carbonization” of waste polyesters into hierarchically porous carbon for high-performance solar steam evaporation. J Mater Chem A.

[CR119] Zhang H, Zhou X, Shao L, Lü F, He P (2019). Hierarchical porous carbon spheres from low-density polyethylene for high-performance supercapacitors. ACS Sustain Chem Eng.

[CR120] Zhang H, Zhou X, Shao L, Lü F, He P (2021). Upcycling of PET waste into methane-rich gas and hierarchical porous carbon for high-performance supercapacitor by autogenic pressure pyrolysis and activation. Sci Total Environ.

[CR121] Zhang Y, Nahil MA, Wu C, Williams PT (2017). Pyrolysis–catalysis of waste plastic using a nickel–stainless-steel mesh catalyst for high-value carbon products. Environ Technol.

[CR122] Zhang Y, Shen Z, Yu Y, Liu L, Wang G, Chen A (2018). Porous carbon derived from waste polystyrene foam for supercapacitor. J Mater Sci.

[CR123] Zhang Y, Zhu H, Yao D, Williams PT, Wu C, Xu D, Hu Q, Manos G, Yu L, Zhao M, Shearing PR, Brett DJL (2021). Thermo-chemical conversion of carbonaceous wastes for CNT and hydrogen production: a review. Sustain Energy Fuels.

[CR124] Zhang Y, Zhu H, Zhang R, Yu L, Liu Z, Shearing PR, Brett DJL, Williams PT (2022). Study of tire pyrolysis oil model compound structure on carbon nanomaterial production. ACS Sustain Chem Eng.

[CR125] Zhou X, He P, Peng W, Lü F, Shao L, Zhang H (2022). From plastics to methane and carbon spheres: the evolution of pyrolysis products during pyrolysis under autogenic atmosphere. J Anal Appl Pyrolysis.

[CR126] Zhou X, He P, Peng W, Yi S, Lü F, Shao L, Zhang H (2022). Upcycling waste polyvinyl chloride: one-pot synthesis of valuable carbon materials and pipeline-quality syngas via pyrolysis in a closed reactor. J Hazard Mater.

[CR127] Zhuo C, Alves JO, Tenorio JAS, Levendis YA (2012). Synthesis of carbon nanomaterials through up-cycling agricultural and municipal solid wastes. Ind Eng Chem Res.

[CR128] Zhuo C, Hall B, Richter H, Levendis Y (2010). Synthesis of carbon nanotubes by sequential pyrolysis and combustion of polyethylene. Carbon.

[CR129] Zhuo C, Levendis YA (2014). Upcycling waste plastics into carbon nanomaterials: a review. J Appl Polym Sci.

